# Cyclization-Carbonylation-Cyclization Coupling Reaction of Propargyl Ureas with Palladium(II)-Bisoxazoline Catalyst

**DOI:** 10.3390/molecules17089220

**Published:** 2012-08-02

**Authors:** Taichi Kusakabe, Koujiro Kawaguchi, Miya Kawamura, Naohiko Niimura, Rong Shen, Hiroyuki Takayama, Keisuke Kato

**Affiliations:** 1 Faculty of Pharmaceutical Sciences, Toho University, 2-2-1 Miyama, Funabashi, Chiba 274-8510, Japan; Email: taichi.kusakabe@phar.toho-u.ac.jp (T.K.); 2 School of Pharmacy, Nihon Pharmaceutical University, 10281, Komuro, Inamachi, Kita-Adachigun, Saitama 362-0806, Japan

**Keywords:** CCC-coupling, bisoxazoline, palladium, carbonylation, propargyl urea

## Abstract

The cyclization-carbonylation-cyclization coupling reaction (CCC-coupling reaction) of propargyl ureas catalyzed by Pd^II^(box) complexes afforded symmetrical ketones bearing two 2-amino-2-oxazoline groups in good to moderate yields.

## 1. Introduction

**Scheme 1:**
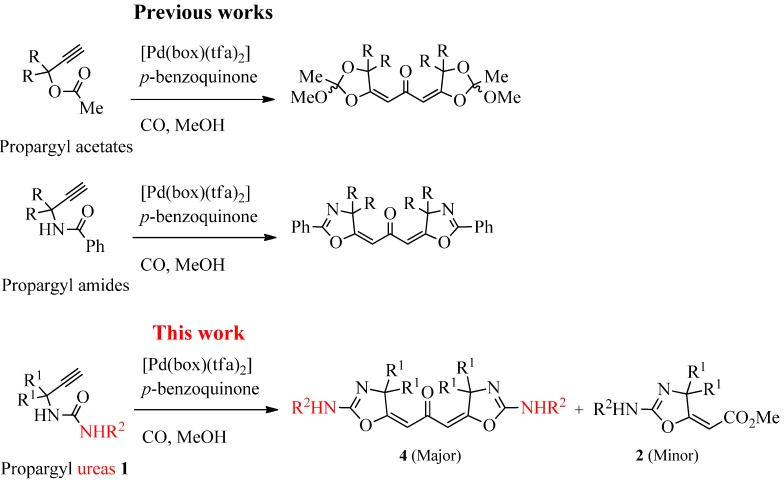
Previous works and this work: CCC-coupling reaction of propargyl acetates, amides and ureas.

Oxazolines appear in numerous medicinally active compounds and natural products [1,2,3]. Among them, 2-amino-2-oxazolines show various interesting pharmacological properties such as anti-hypertensive [4], antidepressant [5], appetite suppressant [6], nitric oxide synthase inhibitor [7] and central nervous system activity [8]. Diarylketones are also frequently found in natural products and pharmaceuticals [9,10]. In addition, they are good precursors for non-steroidal antiestrogen drugs (e.g., tamoxifen) and diarylmethyl compounds [11]. Cascade reactions are important tools for constructing a variety of heterocycles in one step starting from simple compounds [12,13]. Recently, we reported the cyclization-carbonylation-cyclization (CCC)-coupling reaction of propargylic acetates, amides, γ-propynyl-1,3-diketones, *N*-propargylanilines and *o*-alkynylphenols catalyzed by palladium(II)-bisoxazoline (box) complexes (Scheme 1). Symmetrical ketones bearing two cyclic orthoesters [14], oxazolines [14], oxabicyclic groups [15], quinolines [16] and benzofurans [16] were obtained in a one-step reaction.

**Scheme 2:**
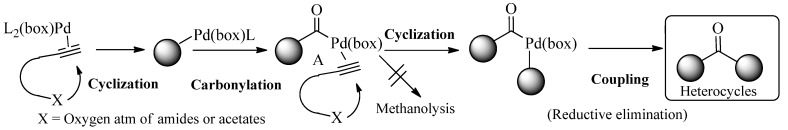
Our concept of a cyclization-carbonylation-cyclization coupling reaction (CCC coupling reaction) of propargylic compounds.

**Scheme 3:**
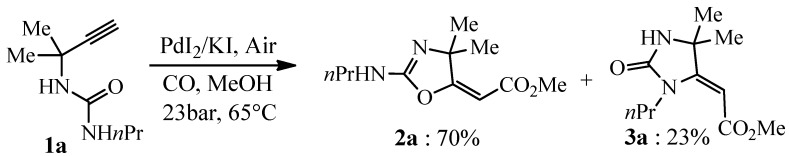
Bacchi *et al*.: PdI_2_-KI catalyzed cyclization-alkoxycarbonylation of **1 [20,21]**.

In these transformations, the triple bond of the substrate coordinates to palladium(II) and undergoes nucleophilic attack by the intramolecular nucleophile X, followed by CO insertion to produce the acyl palladium intermediate **A** (Scheme 2). Coordination of the triple bond of a second molecule induces the second cyclization. Reductive elimination then leads to formation of a ketone bearing two heterocyclic groups. We believe that the box ligand enhances the π-electrophilicity of palladium(II) [14,15,16,17,18,19], and thus promotes coordination of the second triple bond to the acyl palladium intermediate **A**, leading to dimerization. Previously, Bacchi *et al.* reported that the PdI_2_-KI catalyzed cyclization-alkoxycarbonylation of propargyl urea **1a** afforded 2-amino-2-oxazoline derivative **2a** in 70% yield along with imidazoline derivative **3a** in 23% yield (Scheme 3) [20,21]. To extend our concept of the CCC-coupling reaction, we investigated the Pd^II^(box) catalyzed carbonylation reaction of propargyl ureas **1** (Scheme 1).

## 2. Results and Discussion

### 2.1. Optimization Studies

Initially, we selected **1a** as a standard substrate to search for potential catalysts (Table 1). The reaction of **1a** with (CH_3_CN)_2_PdCl_2_ (5 mol %) in the presence of *p-*benzoquinone (1.5 equiv.) in methanol under a carbon monoxide atmosphere (balloon) generated the dimeric ketone **4a** in 46% yield, along with monomeric ester **2a** (36% yield) (Table 1, entry 1). These products were easily separated by chromatography on silica gel. 

**Table 1:** Optimization of reaction condition. 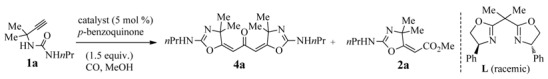

Entry	Catalyst(5 mol %)	Conditions	Yield of4a (%)	Yield of2a (%)
1	(CH_3_CN)_2_PdCl_2_	rt, 24 h	**4a**: 46	**2a**: 36
2	Pd(tfa)_2_	−20 °C, 24 h	**4a**: 24	**2a**: 20
3	[Pd(bipy)Cl_2_]	rt, 24 h	**4a**: 29	**2a**: 13
4	(Ph_3_P)_2_PdCl_2_	rt, 19 h	**4a**: 16	**2a**: 61
5	Pd(PPh_3_)_4_	rt, 24 h	-	**2a**: 44
6	[Pd(**L**)(tfa)_2_]	rt, 24 h	**4a**: 83	**2a**: 17
7	[Pd(**L**)(tfa)_2_]	50 °C, 3 h	**4a**: 61	**2a**: 21
8	[Pd(**L**)(tfa)_2_]	−20 °C, 24 h	**4a**: 30	**2a**: 29
9^ a^	[Pd(**L**)(tfa)_2_]	rt, 24 h	N.R.	
10^ b^	[Pd(**L**)(tfa)_2_]	rt, 24 h	N.R.	

^a^ CH_2_Cl_2 _ was used as solvent; ^b^ DMF was used as solvent.

Pd(tfa)_2_ and 2,2′-bipyridine complex also gave a mixture of products **4a** and **2a** in low yields (Table 1, entries 2–3). The use of phosphine complexes, (Ph_3_P)_2_PdCl_2_ and Pd(PPh_3_)_4_, afforded monomeric ester **2a** as a major product (Table 1, entries 4–5). Next, an attempt was made to use the box complex [Pd(**L**)(tfa)_2_] according to our previous results [14,15,16,17,18,19]. As expected, the reaction occurred smoothly, and the yield of **4a** improved to 83% (Table 1, entry 6). Higher temperatures reduced both the reaction time and the dimer **4a**:monomer **2a** ratio (Table 1, entry 7). Lower temperatures reduced the yields of both products (Table 1, entry 8). Furthermore, CH_2_Cl_2_ and DMF were not suitable as solvents (Table 1, entries 9–10). The structure of the dimeric ketone **4a** was confirmed by comparing the corresponding ^1^H- and ^13^C-NMR data with those of similar oxazolines [22].

### 2.2. Substrate Scope and Limitations

Having optimized the reaction conditions, we examined the reaction of various propargyl ureas **1b**–**j** with the box complex [Pd(**L**)(tfa)_2_] (Table 2).

**Table 2:** CCC-coupling reaction of propargyl ureas **1**. 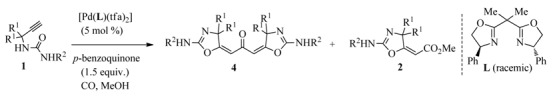

Entry	R^1^	R^2^	Conditions	Yield of4 (%)	Yield of2 (%)
1	Me	*n*Pr	rt, 24 h	**4a**: 83	**2a**: 17
2	Me	Bn	rt, 18 h	**4b**: 73	**2b**: 10
3	Me	*n*Bu	rt, 20 h	**4c**: 61	**2c**: 25
4	Me	Phenethyl	7 °C, 48 h	**4d**: 58	**2d**: 20
5	Me	Cyclohexyl	rt, 24 h	**4e**: 66	**2e**: 30
6	-(CH_2_)_5_-	Phenethyl	rt, 24 h	**4f**: 89	**2f**: 11
7	-(CH_2_)_5_-	Cyclohexyl	rt, 24 h	**4g**: 74	**2g**: 20
8	-(CH_2_)_5_-	Ph	rt, 40 h	**4h**: 24	**2h**: 23
9 ^a^	-(CH_2_)_5_-	H	rt, 48 h	N.R.	
10	H	Phenethyl	rt, 24 h	N.R.	

^a^**1i** was recovered (41%).

The reactions proceeded well for substrates **1a**–**g** bearing two methyl groups in the propargylic position (R^1^ = Me) (Table 2, entries 1–5). The bulky cyclohexyl group in the propargylic position (R^1^ = –(CH_2_)_5_–) did not affect the yields of **4f** and **4g** (Table 2, entries 6 and 7). The alkyl substituent of the terminal nitrogen (R^2^) was found to be important for promoting the cyclization, lower yields were obtained for **1h** (R^2^ = Ph) (Table 2, entry 8). The reaction of unsubstituted **1i** (R^2^ = H) did not proceed (Table 2, entry 9). In addition, the *gem-*dialkyl effect [23] plays a fundamental role for the success of the reaction; the reaction of substrate **1j** having no substituent on the propargylic position did not proceed (Table 2, entry 10).

### 2.3. Preparation of [Pd(L)(tfa)_2_]

**Scheme 4:**
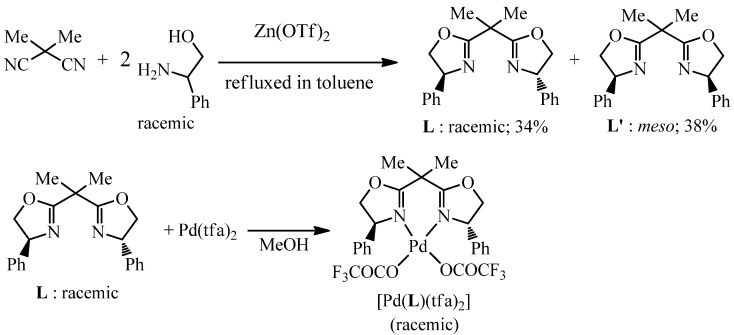
Preparation of [Pd(**L**)(tfa)_2_].

Ligand **L** was prepared according to the previously reported procedure (Scheme 4) [24,25]. Condensation of 2,2-dimethyl-malononitrile with (±)-phenylglycinol using Zn(OTf)_2_ in toluene afforded a mixture of **L** (racemic) and **L′** (*meso*) [18,26]. Chromatographic separation of the mixture gave the compounds in 34% and 38% yields, respectively. The key [Pd(**L**)(tfa)_2_] [28] catalyst was easily obtained as a stable solid by simple filtration from a methanolic mixture of **L** and Pd(tfa)_2_.

## 3. Experimental

### 3.1. General

All melting points were measured on a Yanaco MP-3S micro melting point apparatus and are uncorrected. ^1^H-, ^13^C-NMR and HMBC spectra were recorded on JEOL AL 400 and JEOL Lambda 500 spectrometer spectrometers in CDCl_3_ with Me_4_Si as an internal reference. ^13^C-NMR spectra were recorded at 100 MHz. In the case of CD_2_Cl_2_, solvent peaks were used as a reference (5.32 ppm for ^1^H, and 53.8 ppm for ^13^C). High-resolution mass spectra (HR-MS) were obtained with JEOL GC Mate II, JMS-SX102 and JEOL JMS 600H spectrometer. IR spectra were recorded with JASCO FT/IR-300 spectrometer. All reagents were purchased from commercial sources and used without purification. All evaporations were performed under reduced pressure. For column chromatography, silica gel (Kieselgel 60) was employed. 

### 3.2. Preparation of Substrates *1a–j*

The substrates **1a**–**j** were prepared according to the literature [27]. The substrates **1**, except for **1a** [20] and **1b** [20] are new compounds [22].

### 3.3. General Procedure for the CCC-Coupling Reaction of *1*

A 50-mL two-neck round-bottom flask containing a magnetic stirring bar, substrate **1** (0.5 mmol), *p*-benzoquinone (1.5 mmol) and MeOH (7 mL) was fitted with a rubber septum and a three-way stopcock connected to a balloon filled with carbon monoxide. The apparatus was purged with carbon monoxide by pump-filling via the three-way stopcock. A MeOH (1 mL) suspension of [Pd(**L**)(tfa)_2_] (0.025 mmol) was added to the stirred solution at an appropriate temperature using a syringe. The remaining [Pd(**L**)(tfa)_2_] was washed in MeOH (1 mL) twice. After stirring at the appropriate temperature for a period of time, the mixture was diluted with CH_2_Cl_2_ (50 mL) and washed with 3% NaOH (40 mL). The aqueous layer was extracted with CH_2_Cl_2_ (50 mL) twice and the combined organic layers were dried over MgSO_4_ and concentrated *in vacuo*. The crude product was purified by chromatography on silica gel. The fraction eluted with hexane-AcOEt (30/1–1/1) afforded the monomeric ester **2** and the dimeric ketone **4**. **4** was then precipitated from the reaction mixture and the resulting precipitate was collected by filtration and washed with cold MeOH (1 mL × 2). The filtrate was reprocessed via the above procedure to provide additional products after chromatography.

#### 3.3.1. Dimeric Ketone **4a**

Hexane/AcOEt = 1/1. Colorless needles; mp 186–191 °C; ^1^H-NMR (CDCl_3_) δ 0.95 (6 H, t, *J* = 7.2 Hz), 1.55–1.65 (4 H, m), 1.63 (12 H, s), 3.20 (4 H, t, *J* = 7.2 Hz), 4.11 (2 H, br-s), 5.91 (2 H, s); ^13^C-NMR (CDCl_3_) δ 11.2 (2C), 22.9 (2C), 25.4 (4C), 44.6 (2C), 71.0 (2C), 103.2 (2C), 154.4 (2C), 177.2 (2C), 186.4; IR (KBr): 3203, 3114, 2963, 2877, 1724, 1629, 1370, 1183, 969, 930 cm^−1^; HRMS-EI:*m/z* [M^+^] calcd for C_19_H_30_N_4_O_3_: 362.2318; found: 362.2315.

#### 3.3.2. (2*E*)-Methyl 2-[4,4-dimethyl-2-(propylamino)-5(4*H*)-oxazolylidene]acetate (**2a**)

Hexane/AcOEt = 4/1. Spectral data were identical to those described in the literature [20].

#### 3.3.3. Dimeric Ketone **4b**

Hexane/AcOEt = 1/1. Colorless needles; mp 155–158 °C; ^1^H-NMR (CDCl_3_) δ 1.65 (12 H, s), 4.40 (2 H, s), 5.92 (2 H, s), 7.26–7.36 (10 H, m); ^13^C-NMR (CDCl_3_) δ 25.4 (4C), 46.8 (2C), 70.9 (2C), 103.5 (2C), 127.6 (4C), 127.7 (2C), 128.7 (4C), 137.8 (2C), 154.6 (2C), 176.9 (2C), 186.2; IR (KBr): 2930, 1730, 1630, 1182, 966, 933 cm^−1^; HRMS-EI:*m/z* [M^+^] calcd for C_27_H_30_N_4_O_3_: 458.2318; found: 458.2320.

#### 3.3.4. *(2E)-Methyl 2-[4,4-dimethyl-2-(phenylmethylamino)-5(4H)-oxazolylidene]acetate* (**2b**)

Hexane/AcOEt = 4/1. Spectral data were identical to those described in the literature [20].

#### 3.3.5. Dimeric Ketone **4c**

Hexane/AcOEt = 1/1. Colorless needles; mp 189–191 °C; ^1^H-NMR (CDCl_3_) δ 0.94 (6 H, t, *J* = 7.2 Hz), 1.33–1.59 (8 H, m), 1.63 (12 H, s), 3.23 (4 H, t, *J* = 7.2 Hz), 4.07 (2 H, br-s), 5.91 (2 H, s); ^13^C-NMR (CDCl_3_) δ 13.8 (2C), 19.9 (2C), 25.4 (4C), 31.7(2C), 42.6 (2C), 71.0 (2C), 103.2 (2C), 154.4 (2C), 177.1 (2C), 186.4; IR (KBr): 3206, 2965, 1725, 1627, 1184, 929 cm^−1^; HRMS-EI:*m/z* [M^+^] calcd for C_21_H_34_N_4_O_3_: 390.2631; found: 390.2632.

#### 3.3.6. (2*E*)-Methyl 2-[4,4-dimethyl-2-(butylamino)-5(4*H*)-oxazolylidene]acetate (**2c**)

Hexane/AcOEt = 4/1. Colorless needles; mp 96–99 °C; ^1^H-NMR (CDCl_3_) δ 0.94 (3 H, t, *J* = 7.2 Hz), 1.35–1.43 (2 H, m), 1.52–1.62 (8 H, m), 3.23 (2 H, t, *J* = 7.2 Hz), 3.69 (3 H, s), 4.13 (1 H, br-s), 5.54 (1 H, s); ^13^C-NMR (CDCl_3_) δ 13.7, 19.9, 25.9 (2C), 31.7, 42.6, 51.1, 70.7, 92.6, 154.3, 166.6, 178.5; IR (KBr): 3179, 2967, 1723, 1708, 1689, 1656, 1172, 1095 cm^−1^; HRMS-EI:*m/z* [M^+^] calcd for C_12_H_20_N_2_O_3_: 240.1474; found: 240.1473.

#### 3.3.7. Dimeric Ketone **4d**

Hexane/AcOEt = 1/1. Colorless needles; mp 180–182 °C; ^1^H-NMR (CDCl_3_) δ 1.63 (12 H, s), 2.89 (4 H, t, *J* = 6.8 Hz), 3.51 (4 H, t, *J* = 6.8 Hz), 4.01 (2 H, br-s), 5.87 (2 H, s), 7.19–7.33 (10 H, m); ^13^C-NMR (CDCl_3_) δ 25.4 (4C), 35.4 (2C), 43.7 (2C), 71.0 (2C), 103.3 (2C), 126.7 (2C), 128.7 (4C), 128.8 (4C), 138.4 (2C), 154.2 (2C), 177.0 (2C), 186.4; IR (KBr): 3211, 3108, 2942, 1736, 1626, 1180, 966, 931 cm^−1^; HRMS-EI:*m/z* [M^+^] calcd for C_29_H_34_N_4_O_3_: 486.2631; found: 486.2631.

#### 3.3.8. (2*E*)-Methyl 2-[4,4-dimethyl-2-(phenylethylamino)-5(4*H*)-oxazolylidene]acetate (**2d**)

Hexane/AcOEt = 3/1. Brown oil; ^1^H-NMR (CDCl_3_) δ 1.62 (6 H, s), 2.89 (2 H, t, *J* = 6.8 Hz), 3.51 (2 H, t, *J* = 6.8 Hz), 3.68 (3 H, s), 4.35 (1 H, br-s), 5.52 (1 H, s), 7.19–7.31 (5 H, m); ^13^C-NMR (CDCl_3_) δ 25.9 (2C), 35.5, 43.6, 51.1, 70.7, 92.9, 126.7, 128.7 (2C), 128.8 (2C), 138.3, 154.3, 166.5, 178.2; IR (KBr): 3360, 3193, 2972, 1724, 1657, 1106, 1048 cm^−1^; HRMS-EI:*m/z* [M^+^] calcd for C_16_H_20_N_2_O_3_: 288.1474; found: 288.1471.

#### 3.3.9. Dimeric Ketone **4e**

Hexane/AcOEt = 4/1. Colorless needles; mp 243–246 °C; ^1^H-NMR (CDCl_3_) δ 1.19–1.95 (22 H, m), 1.85 (12 H, s), 3.55 (2 H, m), 6.25 (2 H, s); ^13^C-NMR (CDCl_3_) δ 24.5 (4C), 24.7 (4C), 24.7 (2C), 32.7 (4C), 53.2 (2C), 64.9 (2C), 107.0 (2C), 156.4 (2C), 170.5 (2C), 184.1; IR (KBr): 3205, 2933, 2857, 1729, 1625, 1367, 1182, 990, 928 cm^−1^; HRMS-EI:*m/z* [M^+^] calcd for C_25_H_38_N_4_O_3_: 442.2944; found: 442.2943.

#### 3.3.10. (2*E*)-Methyl 2-[4,4-dimethyl-2-(cyclohexylethylamino)-5(4*H*)-oxazolylidene]acetate (**2e**)

Hexane/AcOEt = 5/1. Colorless needles; mp 125–127 °C; ^1^H-NMR (CDCl_3_) δ 1.15–2.06 (10 H, m), 1.62 (6 H, s), 3.46 (1 H, m), 3.69 (3 H, s), 4.04 (1 H, br-s), 5.53 (1 H, s); ^13^C-NMR (CDCl_3_) δ 24.6, 25.5 (2C), 25.9 (2C), 33.3 (2C), 51.0, 51.3, 70.8, 92.4, 153.3, 166.6, 178.5; IR (KBr): 3206, 2934, 2856, 1717, 1658, 1538, 1102 cm^−1^; HRMS-EI:*m/z* [M^+^] calcd for C_14_H_22_N_2_O_3_: 266.1631; found: 266.1633.

#### 3.3.11. Dimeric Ketone **4f**

Hexane/AcOEt = 10/1. Colorless needles; mp 186–190 °C; ^1^H-NMR (CDCl_3_) δ 1.32–1.80 (16 H, m), 2.65–2.72 (4 H, m), 2.89 (4 H, t, *J* = 6.8 Hz), 3.51 (4 H, t, *J* = 6.8 Hz), 4.06 (2 H, br-s), 5.88 (2 H, s), 7.19–7.31 (10 H, m); ^13^C-NMR (CDCl_3_) δ 22.3 (4C), 25.2 (2C), 32.9 (4C), 35.7 (2C), 43.9 (2C), 74.4 (2C), 103.6 (2C), 126.6 (2C), 128.7 (4C), 128.9 (4C), 138.6 (2C), 153.6 (2C), 177.3 (2C), 186.3; IR (KBr): 3418, 2930, 2860, 1720, 1625, 1014, 976, 931 cm^−1^; HRMS-EI:*m/z* [M^+^] calcd for C_35_H_42_N_4_O_3_: 566.3257; found: 566.3260.

#### 3.3.12. Monomeric Ester **2f**

Hexane/AcOEt = 15/1. Colorless needles; mp 104–106 °C; ^1^H-NMR (CDCl_3_) δ 1.37–1.82 (8 H, m), 2.55–2.62 (2 H, m), 2.89 (2 H, t, *J* = 6.8 Hz), 3.51 (2 H, t, *J* = 6.8 Hz), 3.68 (3 H, s), 4.09 (1 H, br-s), 5.52 (1 H, s); ^13^C-NMR (CDCl_3_) δ 22.2 (2C), 25.1, 33.6 (2C), 35.6, 43.9, 51.1, 74.2, 92.6, 126.6, 128.7, 128.8, 138.6, 153.5, 166.7, 179.2; IR (KBr): 3111, 2969, 1732, 1655, 1127, 1065 cm^−1^; HRMS-EI:*m/z* [M^+^] calcd for C_19_H_24_N_2_O_3_: 328.1787; found: 328.1786.

#### 3.3.13. Dimeric Ketone **4g**

Hexane/AcOEt = 15/1. Colorless needles; mp 189–191 °C; ^1^H-NMR (CDCl_3_) δ 1.11–1.79 (32 H, m), 2.00–2.03 (4 H, m), 2.65–2.72 (4 H, m), 3.40–3.47 (2 H, m), 3.94 (2 H, br-s), 5.91 (2 H, s); ^13^C-NMR (CDCl_3_) δ 22.3 (4C), 24.6 (4C), 25.1 (2C), 25.5 (2C), 32.8 (4C), 33.2 (4C), 51.3 (2C), 74.2 (2C), 103.4 (2C), 153.0 (2C), 177.3 (2C), 186.3; IR (KBr): 3430, 2928, 2855, 1710, 1624, 1498, 996, 927 cm^−1^; HRMS-EI:*m/z* [M^+^] calcd for C_31_H_46_N_4_O_3_: 522.3570; found: 522.3568.

#### 3.3.14. Monomeric Estere **2g**

Hexane/AcOEt = 20/1. Colorless needles; mp 133–135 °C; ^1^H-NMR (CDCl_3_) δ 1.12–2.05 (18 H, m), 2.55–2.62 (2 H, m), 3.42–3.50 (1 H, m), 3.69 (3 H, s), 3.96 (1 H, br-s), 5.54 (1 H, s); ^13^C-NMR (CDCl_3_) δ 22.2 (2C), 24.6 (2C), 25.2, 25.6, 33.2 (2C), 33.4 (2C),51.1, 51.4, 74.2, 92.4, 152.8, 166.8, 179.3; IR (KBr): 3358, 2932, 2857, 1707, 1644, 1518, 1125, 1066 cm^−1^; HRMS-EI:*m/z* [M^+^] calcd for C_17_H_2__6_N_2_O_3_: 306.1944; found: 306.1945.

#### 3.3.15. Dimeric Ketone **4h**

Hexane/AcOEt = 10/1. Colorless needles; mp 300 °C; ^1^H-NMR (CDCl_3_) δ 1.46–1.91 (16 H, m), 2.67–2.73 (4 H, m), 6.30 (2 H, s), 7.16–7.56 (10 H, m); ^13^C-NMR (CDCl_3_) δ 21.4 (4C), 23.9 (2C), 31.9 (4C), 69.4 (2C), 108.4 (2C), 122.5 (4C), 127.8 (2C), 129.8 (4C), 133.2 (2C), 155.9 (2C), 169.3 (2C), 183.7; IR (KBr): 3411, 2928, 2859, 1715, 1628, 1603, 1535, 996 cm^−1^; HRMS-EI:*m/z* [M^+^] calcd for C_31_H_34_N_4_O_3_: 510.2631; found: 510.2629.

#### 3.3.16. Dimeric Ketone **2h**

Hexane/AcOEt = 30/1. Colorless needles; mp 121–124 °C; ^1^H-NMR (CD_2_Cl_2_) δ 1.40–1.89 (8 H, m), 2.61–2.68 (2 H, m), 3.68 (3 H, s), 5.62 (1 H, s), 7.03 (1 H, t, *J* = 7.6 Hz), 7.32 (2 H, dd, *J* = 7.6, 8.0 Hz), 7.55 (2 H, d, *J* = 8.0 Hz); ^13^C-NMR (CD_2_Cl_2_) δ 22.7 (2C), 25.6, 33.6 (2C), 51.4, 75.4, 93.4, 118.2 (2C), 122.9, 129.3 (2C), 139.1, 149.5, 166.7, 177.4; IR (KBr): 3298, 2929, 1688, 1605, 1552, 1315, 1151, 1064 cm^−1^; HRMS-EI:*m/z* [M^+^] calcd for C_17_H_20_N_2_O_3_: 300.1474; found: 300.1472.

### 3.4. Preparation of Ligands and Racemic-[Pd(L)(tfa)_2_]

#### 3.4.1. Preparation of **L** and **L**′

To a mixture of dimethylmalononitrile (681 mg, 7.23 mmol) and (±)-phenylglycinol (2.00 g, 14.5 mmol) in anhydrous toluene (160 mL) under Ar was added zinc triflate (2.63 g, 7.23 mmol), and the mixture was refluxed for 3 days. The mixture was allowed to cool, and was then diluted with a saturated NaHCO_3_ aqueous solution (200 mL) and CHCl_3_ (300 mL). After vigorously stirring for 1 h, the layers were separated. The aqueous layer was extracted with CHCl_3_ (50 mL) twice. The combined organic layers were dried with MgSO_4_ and concentrated *in vacuo*. The crude products were purified by column chromatography on silica-gel (70 g). The fraction eluted with hexane/ethyl acetate (7/1) (containing 0.5% Et_3_N) afforded **L′** (914 mg, 38%) [10] and **L** (818 mg, 34%) as pale yellow oils.

#### 3.4.2. Preparation of [Pd(**L**)(tfa)_2_]

To a stirring solution of **L** (307 mg, 0.92 mmol) in MeOH (4 mL) was added Pd(tfa)_2_ (305 mg, 0.92 mmol) in MeOH (3 mL). The [Pd(**L**)(tfa)_2_] was then precipitated from the reaction mixture, and the resulting precipitate was collected by filtration and washed with cold MeOH (1 mL) to give racemic-[Pd(**L**)(tfa)_2_] [28] as a white powder (483 mg, 79%).

## 4. Conclusions

In conclusion, we have presented a cyclization-carbonylation-cyclization coupling reaction (CCC-coupling reaction) of propargyl ureas **1** catalyzed by Pd^II^(box) complexes. Symmetrical ketones possessing two 2-amino-2-oxazoline groups were obtained in moderate to good yields. We believe that the box ligand enhances the π-electrophilicity of palladium(II), and thus promotes coordination of the triple bond (second molecule) to the acyl palladium intermediate **A**, leading to the dimerization reaction. We are currently investigating additional cascade reactions based on the cyclization-carbonylation-cyclization strategy for the synthesis of other types of ketones containing two hetero-cyclic groups. 

## Supplementary Materials

Supplementary materials can be accessed at: http://www.mdpi.com/1420-3049/17/8/9220/s1.
