# Supplementary material for: Cyclization-Carbonylation-Cyclization Coupling Reaction of Propargyl Ureas with Palladium(II)-Bisoxazoline Catalyst
Source: Molecules. 2012 Aug 2;17(8):9220–30. doi: 10.3390/molecules17089220 (PMC6268340; doi:10.3390/molecules17089220)

Electronic Supporting Information

General Information.

All melting points were measured on a Yanaco MP-3S micro melting point apparatus and are uncorrected. ^{1}H, ^{13}C NMR and HMBC spectra were recorded on JEOL AL 400 and JEOL Lambda 500 spectrometer spectrometers in $CDCl_3$ with Me_4Si as an internal reference. ^{13}C NMR spectra were recorded at 100 MHz. High-resolution mass spectra (HR-MS) were obtained with JEOL GC Mate II, JMS-SX102 and JEOL JMS 600H spectrometer. In the case of CD_2Cl_2, solvent peaks were used as a reference (5.32ppm for ^{1}H, and 53.8ppm for ^{13}C). IR spectra were recorded with JASCO FT/IR-300 spectrometer. All reagents were purchased from commercial sources and used without purification. All evaporations were performed under reduced pressure. For column chromatography, silica gel (Kieselgel 60) was employed.

Preparation of substrates 1.

The substrates **1** was prepared according to the literature.[1] The ureas **1** except **1a** and **1b** are new compounds.[2]

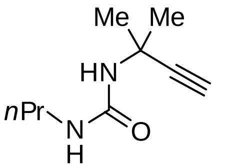

1a[2]

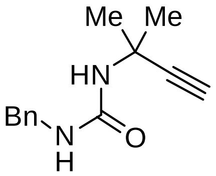

1b[2]

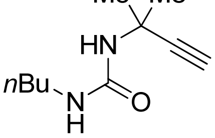

1c : colorless needles; mp 80-83 ℃.

^{1}H-NMR ($CDCl_3$) δ 0.92 (3 H, t, J = 7.2 Hz), 1.33-1.40 (2 H, m), 1.47-1.51 (2 H, m), 1.58 (6 H, s), 2.40 (1H, s), 3.19-3.23 (2 H, m), 4.93 (1 H, br-s), 5.32 (1 H, br-s); ^{13}C-NMR ($CDCl_3$) δ 13.8, 20.1, 30.2 (2C), 32.3, 40.0, 46.7, 70.1, 87.7, 157.6.

IR (KBr): 3360, 3241, 2968, 2105, 1632 cm^{-1}.

HRMS-EI:*m/z* [M^+] calcd for $C_{10}H_{18}N_2O$: 182.1419; found: 182.1418.

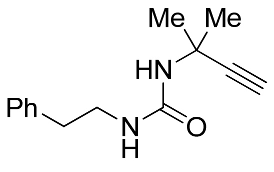

1d : colorless needles; mp 111-113 ℃.

^{1}H-NMR (CDCl$_3$) δ 1.52 (6 H, s), 2.23 (1H, s), 2.82 (2 H, t, J = 7.0 Hz), 3.46-3.51 (2 H, m), 4.76 (1 H, br-s), 5.22 (1 H, br-s), 7.20-7.31 (5 H, m); ^{13}C-NMR (CDCl$_3$) δ 30.0 (2C), 36.2, 41.4, 46.6, 70.3, 87.2, 126.3, 128.5 (2C), 128.9 (2C), 139.2, 159.3.

IR (KBr): 3336, 3284, 2103, 1636, 1568, 1282, 1263, 642 cm^{-1}.

HRMS-EI:*m/z* [M$^+$] calcd for $C_{14}H_{18}N_2O$: 230.1419; found: 230.1415.

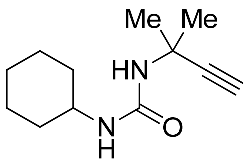

1e : colorless needles; mp 160-164 ℃.

^{1}H-NMR (CDCl$_3$) δ 1.15-1.91 (10 H, m), 1.58 (6H, s), 2.43 (1 H, s), 3.61-3.67 (1 H, m), 4.60 (1 H, s), 5.15 (1 H, d, J = 6.8 Hz); ^{13}C-NMR (CDCl$_3$) δ 24.8 (2C), 25.6, 30.2 (2C), 33.7 (2C), 46.7, 48.8, 70.5, 87.5, 156.7.

IR (KBr): 3317, 3249, 2924, 2106, 1629, 1557, 695 cm^{-1}.

HRMS-EI:*m/z* [M$^+$] calcd for $C_{12}H_{20}N_2O$: 208.1576; found: 208.1572.

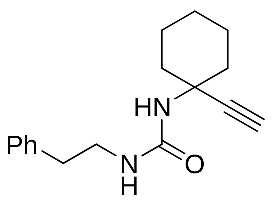

1f : colorless needles; mp 126-129 ℃.

^{1}H-NMR (CDCl$_3$) δ 1.12-2.03 (10 H, m), 2.28 (1H, s), 2.83 (2 H, t, J = 6.8 Hz), 3.50 (2 H, q, J = 6.8 Hz), 4.53 (1 H, br-s), 5.25 (1 H, br-s), 7.20-7.31 (5H, m); ^{13}C-NMR (CDCl$_3$) δ 22.3 (2C), 25.2, 36.1, 38.1 (2C), 41.5, 50.9, 73.2, 85.6, 126.3, 128.5 (2C), 129.0 (2C), 139.4, 157.2.

IR (KBr): 3353, 3235, 2928, 2100, 1628, 1565 cm^{-1}.

HRMS-EI:*m/z* [M$^+$] calcd for $C_{17}H_{22}N_2O$: 270.1732; found: 270.1730.

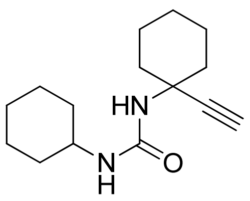

1g : colorless needles; mp 111-113 ℃.

^{1}H-NMR (CDCl$_3$) δ 1.16-2.11 (20 H, m), 2.52 (1H, s), 3.64-3.69 (1 H, m), 4.57 (1 H, s), 5.25 (1 H, d, J = 7.2 Hz); ^{13}C-NMR (CDCl$_3$) δ 22.4 (2C), 24.8 (2C), 25.2, 25.7, 33.7 (2C), 38.2 (2C), 48.8, 50.9, 73.2, 86.0, 156.6.

IR (KBr): 3308, 3278, 2931, 2854, 2103, 1637, 1560, 1254, 637 cm^{-1}.

HRMS-EI:*m/z* [M^+] calcd for $C_{15}H_{24}N_2O$: 248.1889; found: 248.1890.

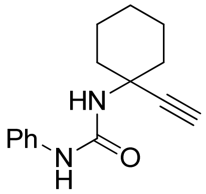

1h : colorless needles; mp 146-150 ℃.

^{1}H-NMR ($CDCl_3$) δ 1.27-1.68 (8 H, m), 2.14-2.15 (2H, m), 2.54 (1 H, s), 5.00 (1 H, br-s), 7.02-7.05 (1 H, m), 7.21 (1 H, br-s), 7.25-7.36 (4 H, m); ^{13}C-NMR ($CDCl_3$) δ 22.4 (2C), 25.2, 38.0 (2C), 51.3, 73.0, 85.9, 120.3 (2C), 123.4, 129.1 (2C), 138.7, 154.7.

IR (KBr): 3347, 3305, 2934, 2859, 2109, 1656, 1603, 1554, 1500, 1312, 1242 cm^{-1}.

HRMS-EI:*m/z* [M^+] calcd for $C_{15}H_{18}N_2O$: 242.1419; found: 242.1420.

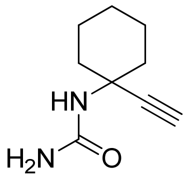

1i : colorless needles; mp 109-113℃.

^{1}H-NMR ($CDCl_3$) δ 1.28-1.67 (8 H, m), 2.11-2.13 (2H, m), 2.51 (1 H, s), 5.18 (2 H, br-s), 5.27 (1 H, br-s); ^{13}C-NMR ($CDCl_3$) δ 22.4 (2C), 25.2, 37.9 (2C), 51.1, 73.1, 85.7, 158.4.

IR (KBr): 3437, 3314, 2936, 2113, 1661, 1610, 1550, 1362 cm^{-1}.

HRMS-EI:*m/z* [M^+] calcd for $C_9H_{14}N_2O$: 166.1106; found: 166.1107.

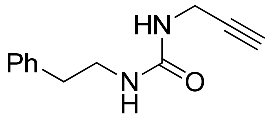

1j : colorless needles; mp 96-99 ℃.

^{1}H-NMR ($CDCl_3$) δ 2.16 (1 H, t, *J* = 2.4 Hz), 2.79 (2 H, t, *J* = 6.8 Hz), 3.39-3.44 (2 H, m), 3.90 (2 H, dd, *J* = 2.4, 5.6 Hz), 5.03 (1 H, br-s), 5.12 (1 H, br-s), 7.17-7.30 (5H, m); ^{13}C-NMR ($CDCl_3$) δ 30.0, 36.4, 41.7, 71.0, 80.8, 126.4, 128.6 (2C), 128.8 (2C), 139.1, 157.9.

IR (KBr): 3353, 3322, 3277, 2116, 1620, 1594 cm^{-1}.

HRMS-EI:*m/z* [M^+] calcd for $C_{12}H_{14}N_2O$: 202.1106; found: 202.1103.

General procedure for the CCC-coupling reaction of 1

A 50-mL two-neck round-bottom flask containing a magnetic stirring bar, substrate **1** (0.5 mmol), *p*-benzoquinone (1.5 mmol) and MeOH (7 mL) was fitted with a rubber septum and a three-way stopcock connected to a balloon filled with carbon monoxide. The apparatus was purged with carbon monoxide by pump-filling via the three-way stopcock. A MeOH (1 mL) suspension of [Pd(**L**)$(tfa)_2$] (0.025 mmol) was added to the stirred solution at an appropriate temperature using a syringe. The remaining [Pd(**L**)$(tfa)_2$] was washed in MeOH (1 mL) twice. After stirring at the appropriate temperature for a

period of time, the mixture was diluted with CH_2Cl_2 (50 mL) and washed with 3% NaOH (40 mL). The aqueous layer was extracted with CH_2Cl_2 (50 mL) twice and the combined organic layers were dried over $MgSO_4$ and concentrated *in vacuo*. The crude product was purified by chromatography on silica gel. The fraction eluted with hexane-AcOEt (10/1-1/2) afforded the dimeric ketone **4**. **4** was then precipitated from the reaction mixture and the resulting precipitate was collected by filtration and washed with cold MeOH (1 mL × 2). The filtrate was reprocessed via the above procedure to provide additional products after chromatography.

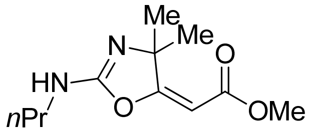

2a : Spectral data were identical to those described in the literature.[2]

The structure of the dimeric ketone **4a** was confirmed by comparing the ^{1}H and ^{13}C-NMR data with those of similar oxazolines **A**, **B** and **C**.[3]

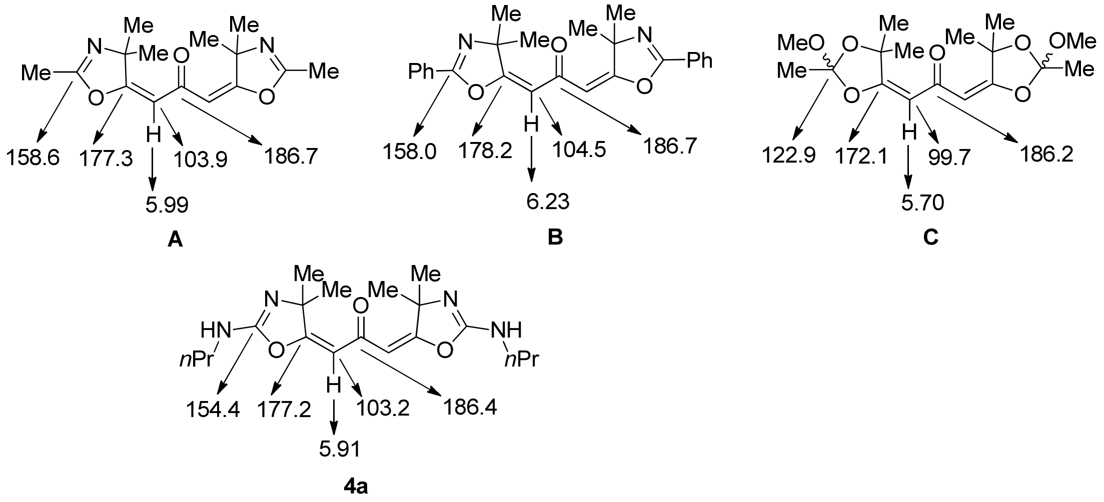

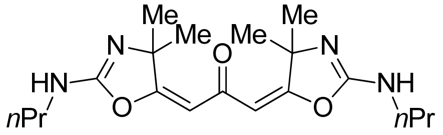

4a : colorless needles; mp 186-191 ℃.

^{1}H-NMR ($CDCl_3$) δ 0.95 (6 H, t, J = 7.2 Hz), 1.55-1.65 (4 H, m), 1.63 (12 H, s), 3.20 (4 H, t, J = 7.2 Hz), 4.11 (2 H, br-s), 5.91 (2 H, s); ^{13}C-NMR ($CDCl_3$) δ 11.2 (2C), 22.9 (2C), 25.4 (4C), 44.6 (2C), 71.0 (2C), 103.2 (2C), 154.4 (2C), 177.2 (2C), 186.4

IR (KBr): 3203, 3114, 2963, 2877, 1724, 1629, 1370, 1183, 969, 930 cm^{-1}.

HRMS-EI:*m/z* [M^+] calcd for $C_{19}H_{30}N_4O_3$: 362.2318; found: 362.2315.

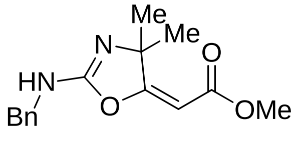

2b : Spectral data were identical to those described in the literature.[2]

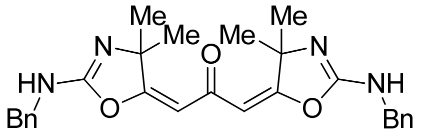

4b : colorless needles; mp 155-158℃

^{1}H-NMR (CDCl$_3$) δ 1.65 (12 H, s), 4.40 (2 H, s), 5.92 (2 H, s), 7.26-7.36 (10 H, m) ; ^{13}C-NMR (CDCl$_3$) δ 25.4 (4C), 46.8 (2C), 70.9 (2C), 103.5 (2C), 127.6 (4C), 127.7 (2C), 128.7 (4C), 137.8 (2C), 154.6 (2C), 176.9 (2C), 186.2

IR (KBr): 2930, 1730, 1630, 1182, 966, 933 cm^{-1}

HRMS-EI:*m/z* [M^{+}] calcd for $C_{27}H_{30}N_4O_3$: 458.2318; found: 458.2320.

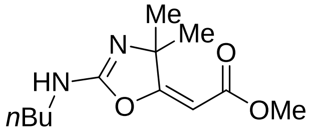

2c : colorless needles; mp 96-99 ℃.

^{1}H-NMR (CDCl$_3$) δ 0.94 (3 H, t, J = 7.2 Hz), 1.35-1.43 (2 H, m), 1.52-1.62 (8 H, m), 3.23 (2 H, t, J = 7.2 Hz), 3.69 (3 H, s), 4.13 (1 H, br-s), 5.54 (1 H, s); ^{13}C-NMR (CDCl$_3$) δ 13.7, 19.9, 25.9 (2C), 31.7, 42.6, 51.1, 70.7, 92.6, 154.3, 166.6, 178.5.

IR (KBr): 3179, 2967, 1723, 1708, 1689, 1656, 1172, 1095 cm^{-1}.

HRMS-EI:*m/z* [M^{+}] calcd for $C_{12}H_{20}N_2O_3$: 240.1474; found: 240.1473.

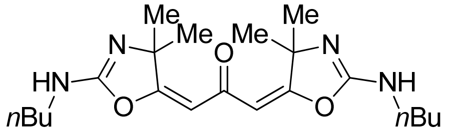

4c : colorless needles; mp 189-191 ℃.

^{1}H-NMR (CDCl$_3$) δ 0.94 (6 H, t, J = 7.2 Hz), 1.33-1.59 (8 H, m), 1.63 (12 H, s), 3.23 (4 H, t, J = 7.2 Hz), 4.07 (2 H, br-s), 5.91 (2 H, s); ^{13}C-NMR (CDCl$_3$) δ 13.8 (2C), 19.9 (2C), 25.4 (4C), 31.7(2C), 42.6 (2C), 71.0 (2C), 103.2 (2C), 154.4 (2C), 177.1 (2C), 186.4

IR (KBr): 3206, 2965, 1725, 1627, 1184, 929 cm^{-1}.

HRMS-EI:*m/z* [M^{+}] calcd for $C_{21}H_{34}N_4O_3$: 390.2631; found: 390.2632.

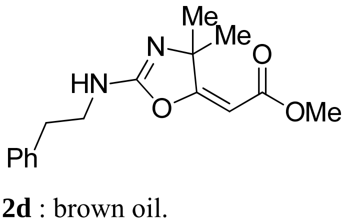

2d : brown oil.

^{1}H-NMR (CDCl$_3$) δ 1.62 (6 H, s), 2.89 (2 H, t, J = 6.8 Hz), 3.51 (2 H, t, J = 6.8 Hz), 3.68 (3 H, s), 4.35 (1 H, br-s), 5.52 (1 H, s), 7.19-7.31 (5 H, m); ^{13}C-NMR (CDCl$_3$) δ 25.9 (2C), 35.5, 43.6, 51.1, 70.7, 92.9, 126.7, 128.7 (2C), 128.8 (2C), 138.3, 154.3, 166.5, 178.2.

IR (KBr): 3360, 3193, 2972, 1724, 1657, 1106, 1048 cm^{-1}.

HRMS-EI:*m/z* [M^{+}] calcd for $C_{16}H_{20}N_2O_3$: 288.1474; found: 288.1471.

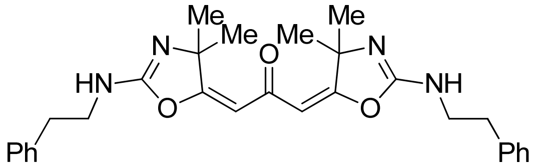

4d : colorless needles; mp 180-182 ℃.

^{1}H-NMR (CDCl$_3$) δ 1.63 (12 H, s), 2.89 (4 H, t, J = 6.8 Hz), 3.51 (4 H, t, J = 6.8 Hz), 4.01 (2 H, br-s), 5.87 (2 H, s), 7.19-7.33 (10 H, m); ^{13}C-NMR (CDCl$_3$) δ 25.4 (4C), 35.4 (2C), 43.7 (2C), 71.0 (2C), 103.3 (2C), 126.7 (2C), 128.7 (4C), 128.8 (4C), 138.4 (2C), 154.2 (2C), 177.0 (2C), 186.4.

IR (KBr): 3211, 3108, 2942, 1736, 1626, 1180, 966, 931 cm^{-1}.

HRMS-EI:*m/z* [M$^+$] calcd for $C_{29}H_{34}N_4O_3$: 486.2631; found: 486.2631.

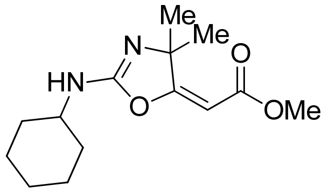

2e : colorless needles; mp 125-127 ℃.

^{1}H-NMR (CDCl$_3$) δ 1.15-2.06 (10 H, m), 1.62 (6 H, s), 3.46 (1 H, m), 3.69 (3 H, s), 4.04 (1 H, br-s), 5.53 (1 H, s); ^{13}C-NMR (CDCl$_3$) δ 24.6, 25.5 (2C), 25.9 (2C), 33.3 (2C), 51.0, 51.3, 70.8, 92.4, 153.3, 166.6, 178.5.

IR (KBr): 3206, 2934, 2856, 1717, 1658, 1538, 1102 cm^{-1}.

HRMS-EI:*m/z* [M$^+$] calcd for $C_{14}H_{22}N_2O_3$: 266.1631; found: 266.1633.

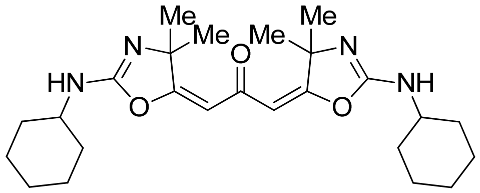

4e : colorless needles; mp 243-246 ℃.

^{1}H-NMR (CDCl$_3$) δ 1.19-1.95 (22 H, m), 1.85 (12 H, s), 3.55 (2 H, m), 6.25 (2 H, s); ^{13}C-NMR (CDCl$_3$) δ 24.5 (4C), 24.7 (4C), 24.7 (2C), 32.7 (4C), 53.2 (2C), 64.9 (2C), 107.0 (2C), 156.4 (2C), 170.5 (2C), 184.1.

IR (KBr): 3205, 2933, 2857, 1729, 1625, 1367, 1182, 990, 928 cm^{-1}.

HRMS-EI:*m/z* [M$^+$] calcd for $C_{25}H_{38}N_4O_3$: 442.2944; found: 442.2943.

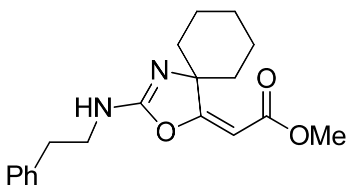

2f : colorless needles; mp 104-106 ℃.

^{1}H-NMR (CDCl$_3$) δ 1.37-1.82 (8 H, m), 2.55-2.62 (2 H, m), 2.89 (2 H, t, J = 6.8 Hz), 3.51 (2 H, t, J = 6.8 Hz), 3.68 (3 H, s), 4.09 (1 H, br-s), 5.52 (1 H, s); ^{13}C-NMR (CDCl$_3$) δ 22.2 (2C), 25.1, 33.6 (2C), 35.6, 43.9, 51.1, 74.2, 92.6, 126.6, 128.7, 128.8, 138.6, 153.5, 166.7, 179.2..

IR (KBr): 3111, 2969, 1732, 1655, 1127, 1065 cm^{-1}.

HRMS-EI:*m/z* [M$^+$] calcd for $C_{19}H_{24}N_2O_3$: 328.1787; found: 328.1786.

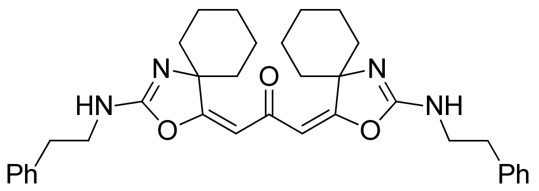

4f : colorless needles; mp 186-190 ℃.

^{1}H-NMR ($CDCl_3$) δ 1.32-1.80 (16 H, m), 2.65-2.72 (4 H, m), 2.89 (4 H, t, J = 6.8 Hz), 3.51 (4 H, t, J = 6.8 Hz), 4.06 (2 H, br-s), 5.88 (2 H, s), 7.19-7.31 (10 H, m); ^{13}C-NMR ($CDCl_3$) δ 22.3 (4C), 25.2 (2C), 32.9 (4C), 35.7 (2C), 43.9 (2C), 74.4 (2C), 103.6 (2C), 126.6 (2C), 128.7 (4C), 128.9 (4C), 138.6 (2C), 153.6 (2C), 177.3 (2C), 186.3.

IR (KBr): 3418, 2930, 2860, 1720, 1625, 1014, 976, 931 cm^{-1}.

HRMS-EI:*m/z* [M^{+}] calcd for $C_{35}H_{42}N_4O_3$: 566.3257; found: 566.3260.

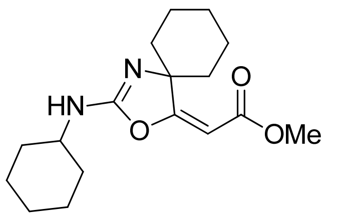

2g :colorless needles; mp 133-135 ℃.

^{1}H-NMR ($CDCl_3$) δ 1.12-2.05 (18 H, m), 2.55-2.62 (2 H, m), 3.42-3.50 (1 H, m), 3.69 (3 H, s), 3.96 (1 H, br-s), 5.54 (1 H, s); ^{13}C-NMR ($CDCl_3$) δ 22.2 (2C), 24.6 (2C), 25.2, 25.6, 33.2 (2C), 33.4 (2C),51.1, 51.4, 74.2, 92.4, 152.8, 166.8, 179.3.

IR (KBr): 3358, 2932, 2857, 1707, 1644, 1518, 1125, 1066 cm^{-1}.

HRMS-EI:*m/z* [M^{+}] calcd for $C_{17}H_{26}N_2O_3$: 306.1944; found: 306.1945.

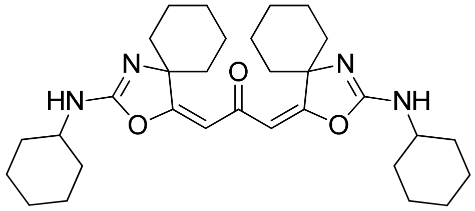

4g : colorless needles; mp 189-191 ℃.

^{1}H-NMR ($CDCl_3$) δ 1.11-1.79 (32 H, m), 2.00-2.03 (4 H, m), 2.65-2.72 (4 H, m), 3.40-3.47 (2 H, m), 3.94 (2 H, br-s), 5.91 (2 H, s); ^{13}C-NMR ($CDCl_3$) δ 22.3 (4C), 24.6 (4C), 25.1 (2C), 25.5 (2C), 32.8 (4C), 33.2 (4C), 51.3 (2C), 74.2 (2C), 103.4 (2C), 153.0 (2C), 177.3 (2C), 186.3.

IR (KBr): 3430, 2928, 2855, 1710, 1624, 1498, 996, 927 cm^{-1}.

HRMS-EI:*m/z* [M^{+}] calcd for $C_{31}H_{46}N_4O_3$: 522.3570; found: 522.3568.

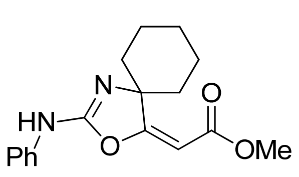

2h : colorless needles; mp 121-124 ℃.

^{1}H-NMR ($CDCl_3$) δ 1.42-1.89 (8 H, m), 2.62-2.70 (2 H, m), 3.71 (3 H, s), 5.62 (1 H, s), 6.23 (1H, br-s), 7.03 (1 H, t, J = 7.6 Hz), 7.32 (2 H, t, J = 7.6 Hz), 7.51 (2 H, d, J = 7.6 Hz); ^{13}C-NMR (CD_2Cl_2) δ 22.7 (2C), 25.6, 33.6 (2C), 51.4, 75.4, 93.4,

118.2 (2C), 122.9, 129.3 (2C), 139.1, 149.5, 166.7, 177.4.

IR (KBr): 3298, 2929, 1688, 1605, 1552, 1315, 1151, 1064 cm^{-1}.

HRMS-EI:*m/z* [M^+] calcd for $C_{17}H_{20}N_2O_3$: 300.1474; found: 300.1472.

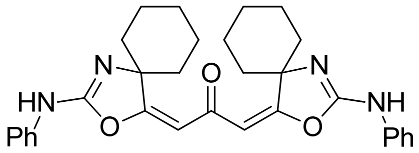

4h : colorless needles; mp 300 ℃.

^{1}H-NMR ($CDCl_3$) δ 1.46-1.91 (16 H, m), 2.67-2.73 (4 H, m), 6.30 (2 H, s), 7.16-7.56 (10 H, m), 8.65 (2H, br-s); ^{13}C-NMR ($CDCl_3$) δ 21.4 (4C), 23.9 (2C), 31.9 (4C), 69.4 (2C), 108.4 (2C), 122.5 (4C), 127.8 (2C), 129.8 (4C), 133.2 (2C), 155.9 (2C), 169.3 (2C), 183.7

IR (KBr): 3411, 2928, 2859, 1715, 1628, 1603, 1535, 996 cm^{-1}.

HRMS-EI:*m/z* [M^+] calcd for $C_{31}H_{34}N_4O_3$: 510.2631; found: 510.2629.

Ref

1) F. E. Michael, P. A. Sibbald, B. M. Cochran, *Org. Lett.* **2008**, *10*, 793.

2) A. Bacchi, G. P. Chiusoli, M. Costa, C. Sani, B. Gabriele, G. Salerno, *J. Organomet. Chem.* **1998**, *562*, 35.

1c proton

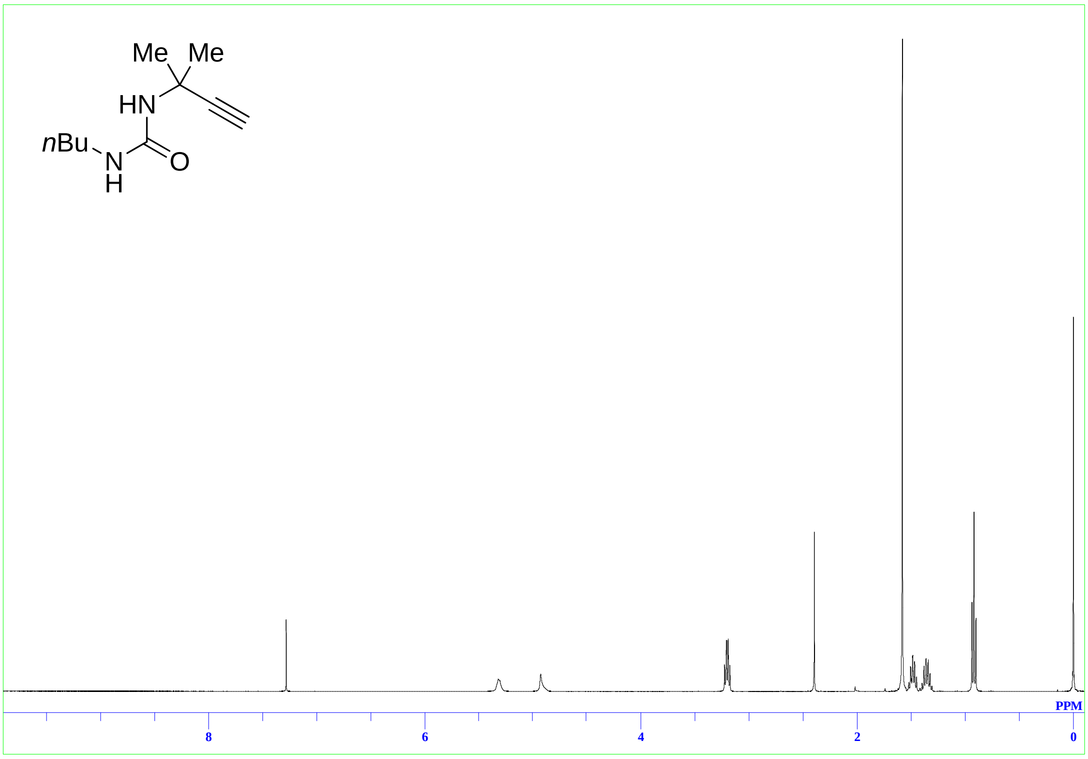

1c carbon

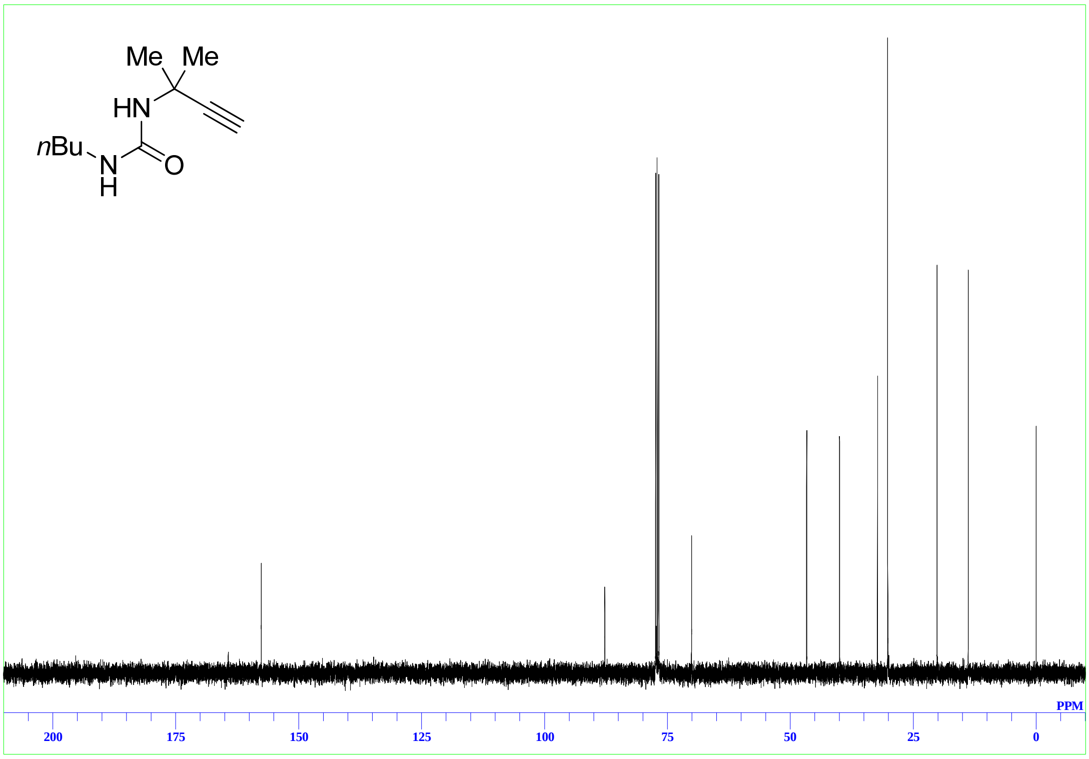

1d proton

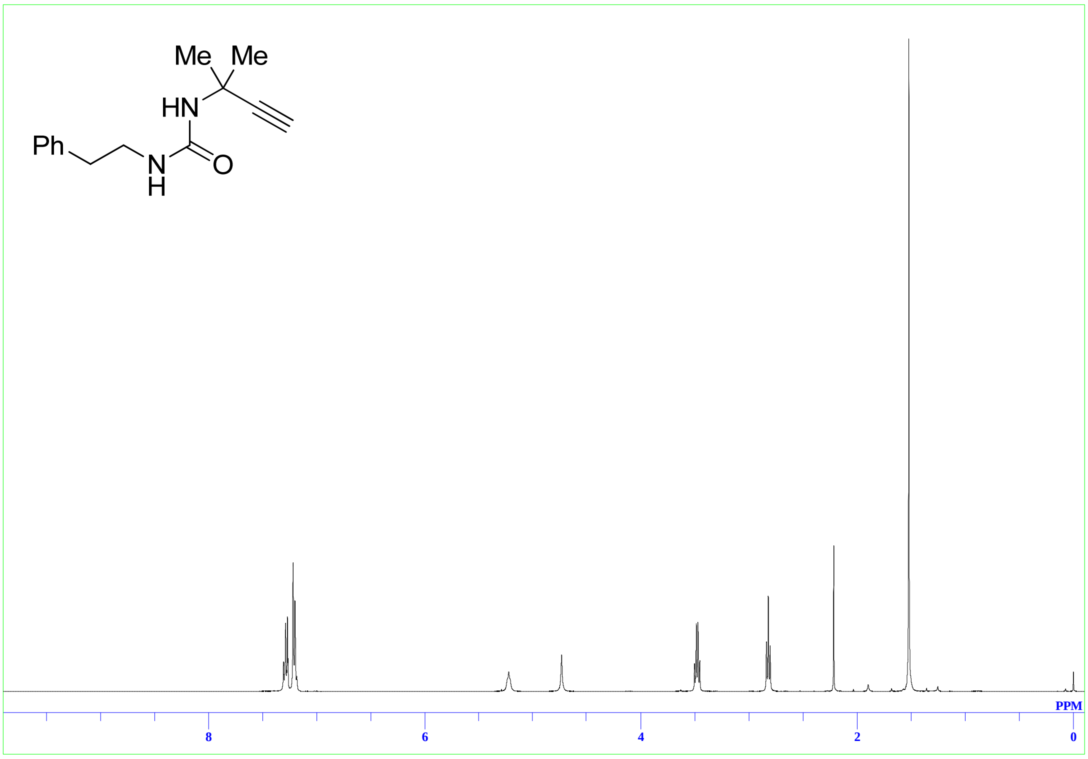

1d carbon

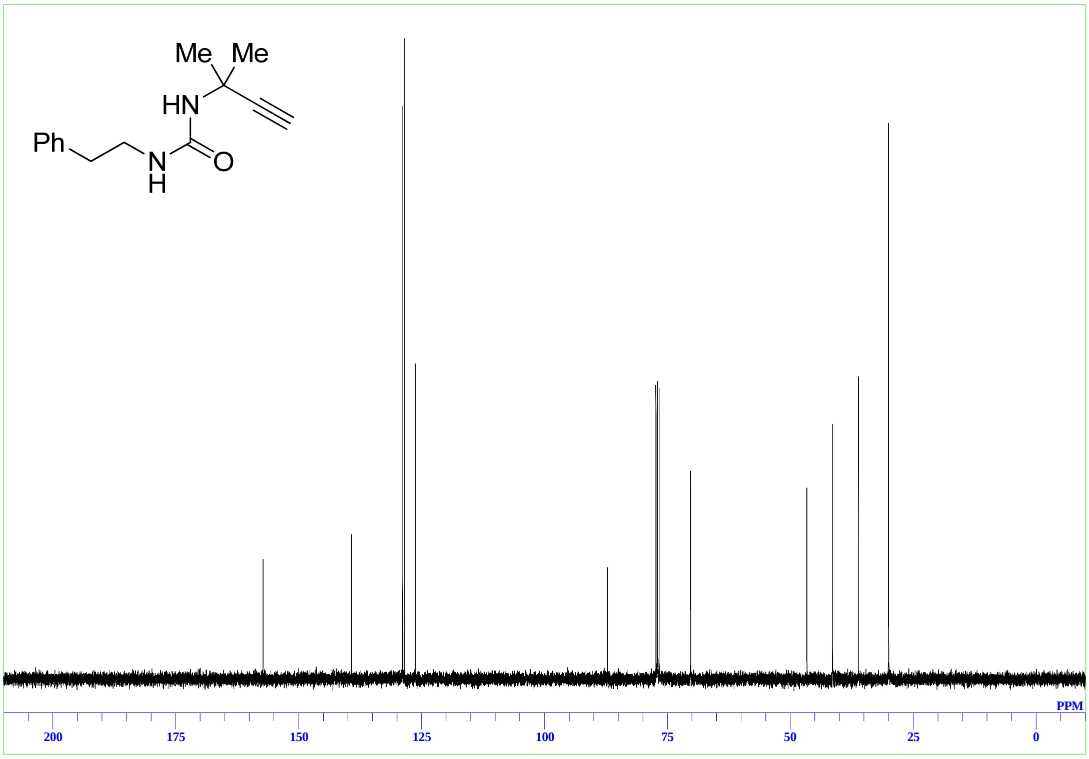

1e proton

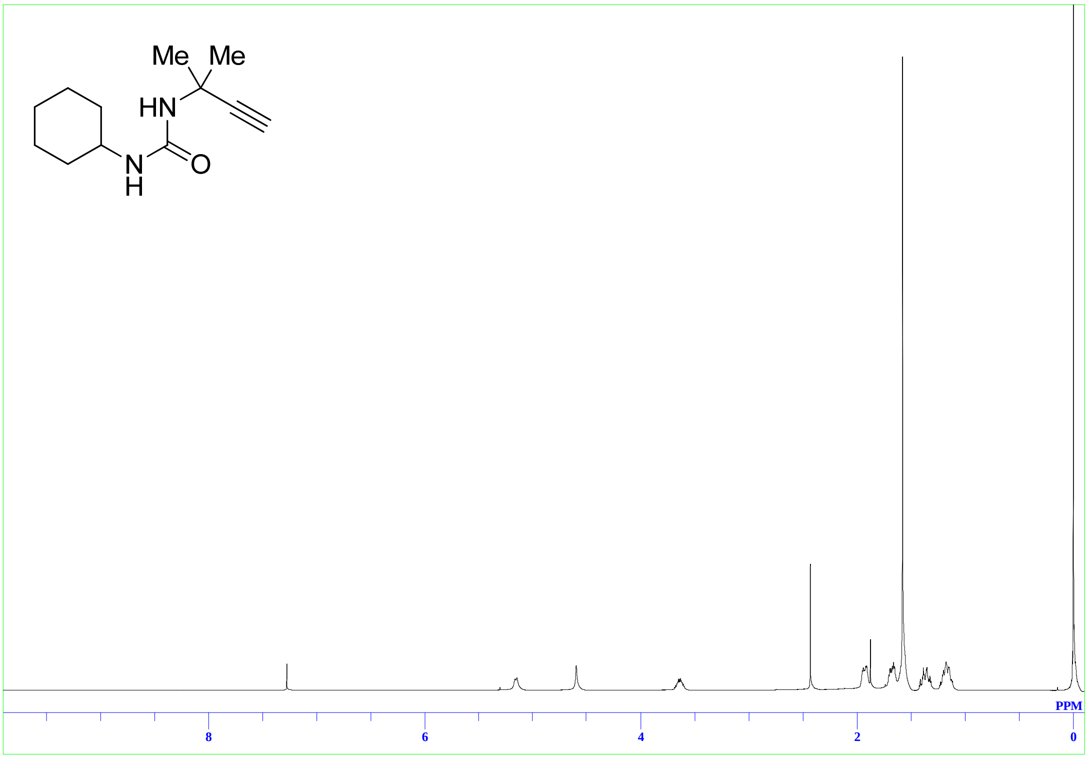

1e carbon

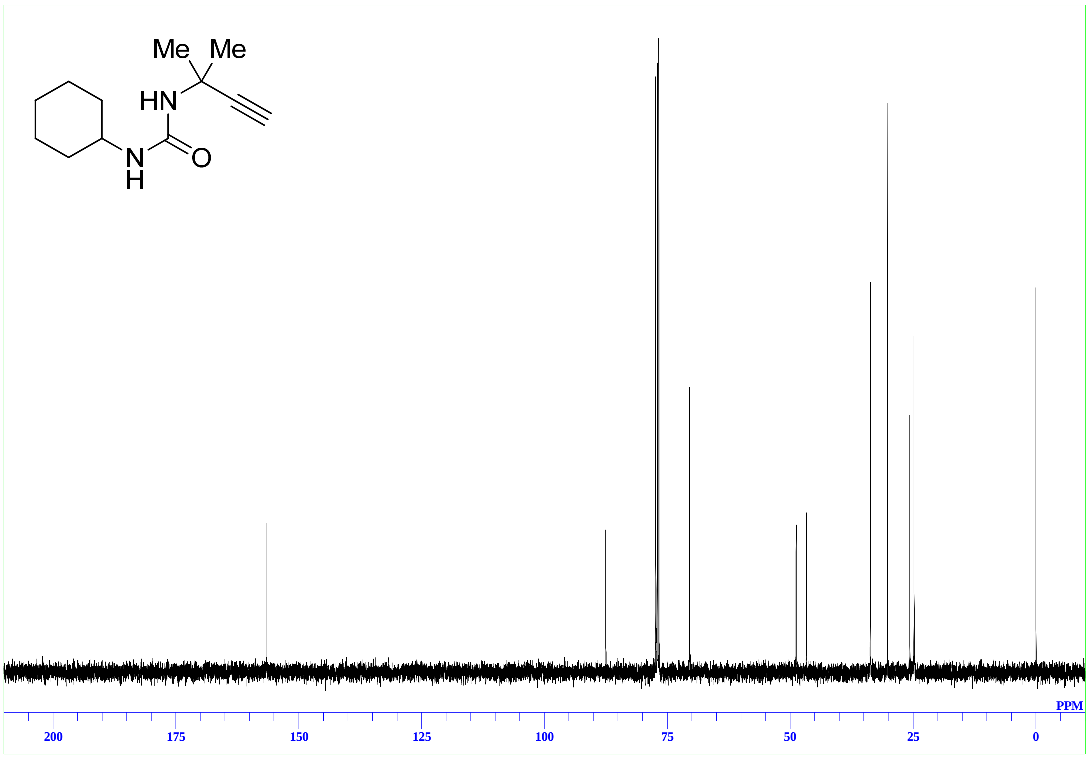

1f proton

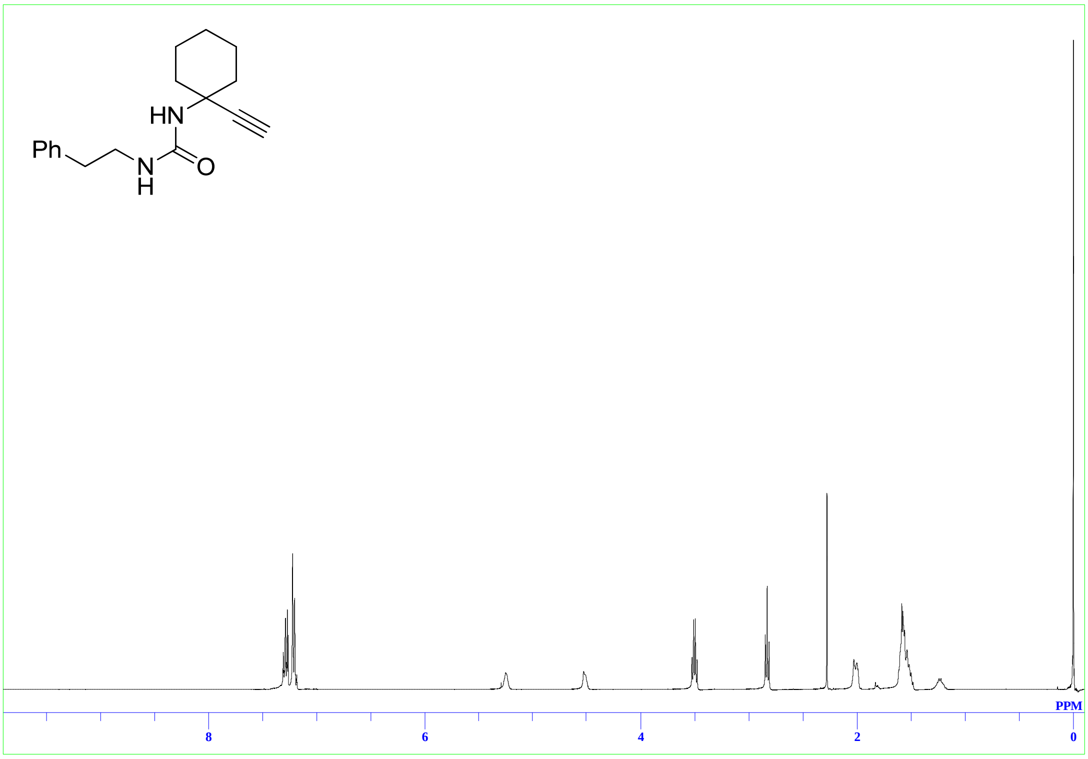

1f carbon

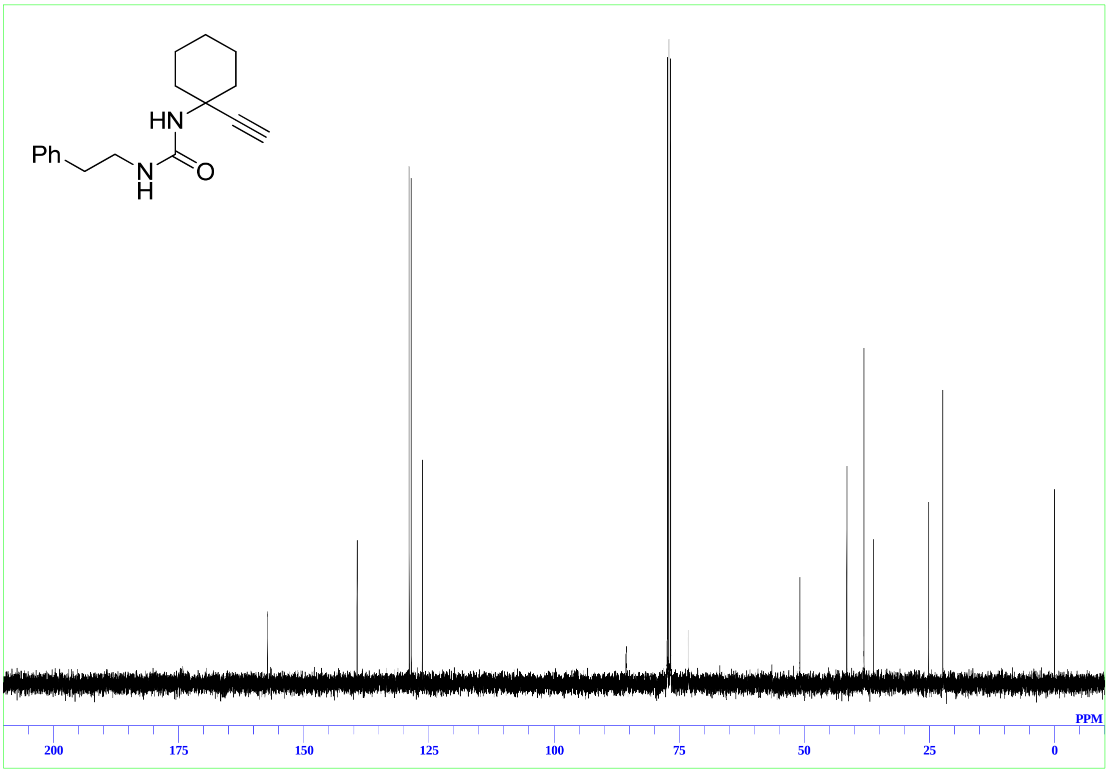

1g proton

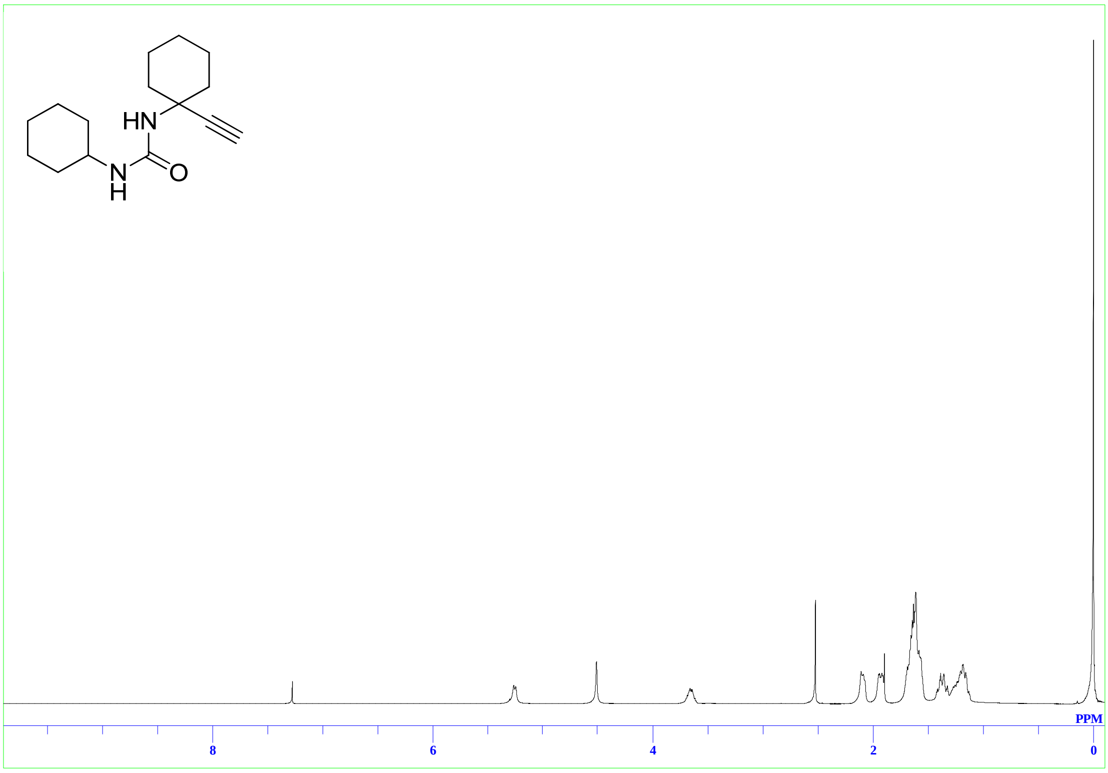

1g carbon

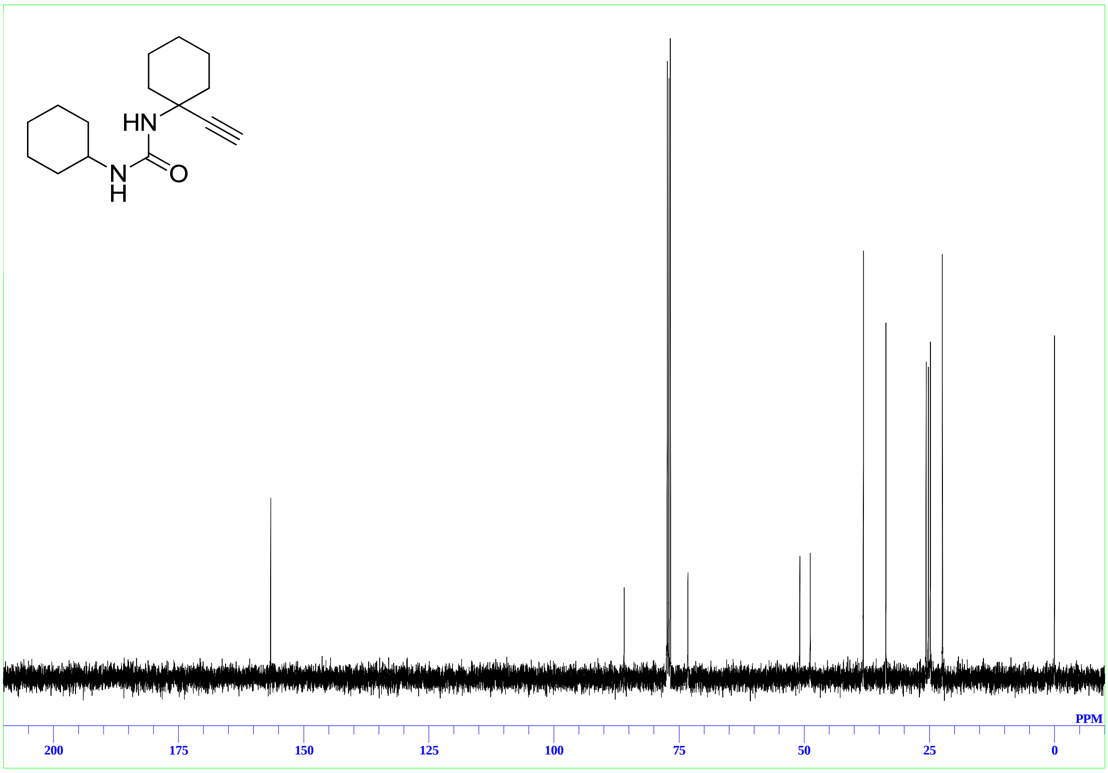

1h proton

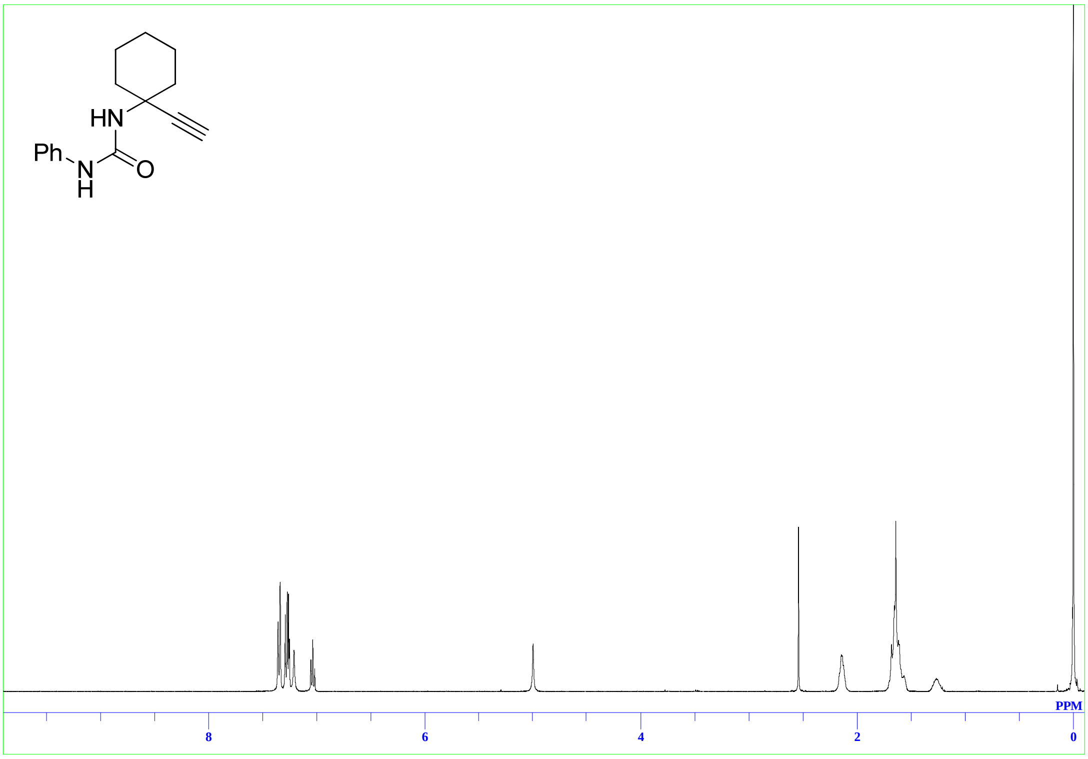

1h carbon

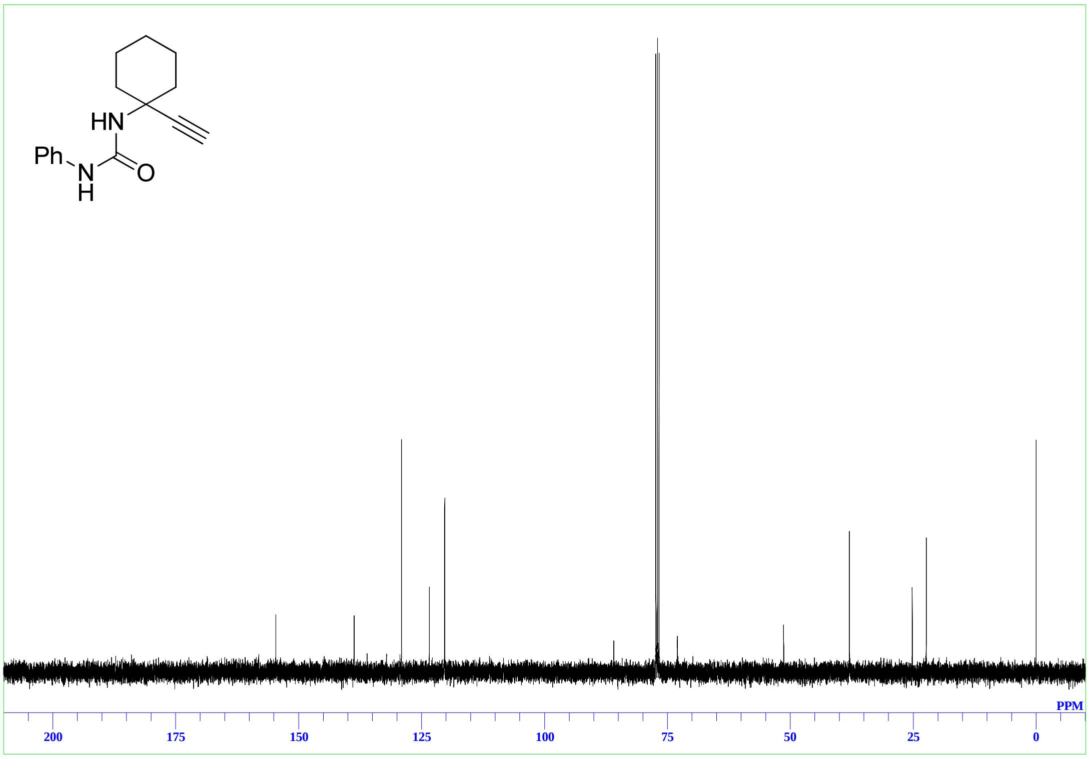

1i proton

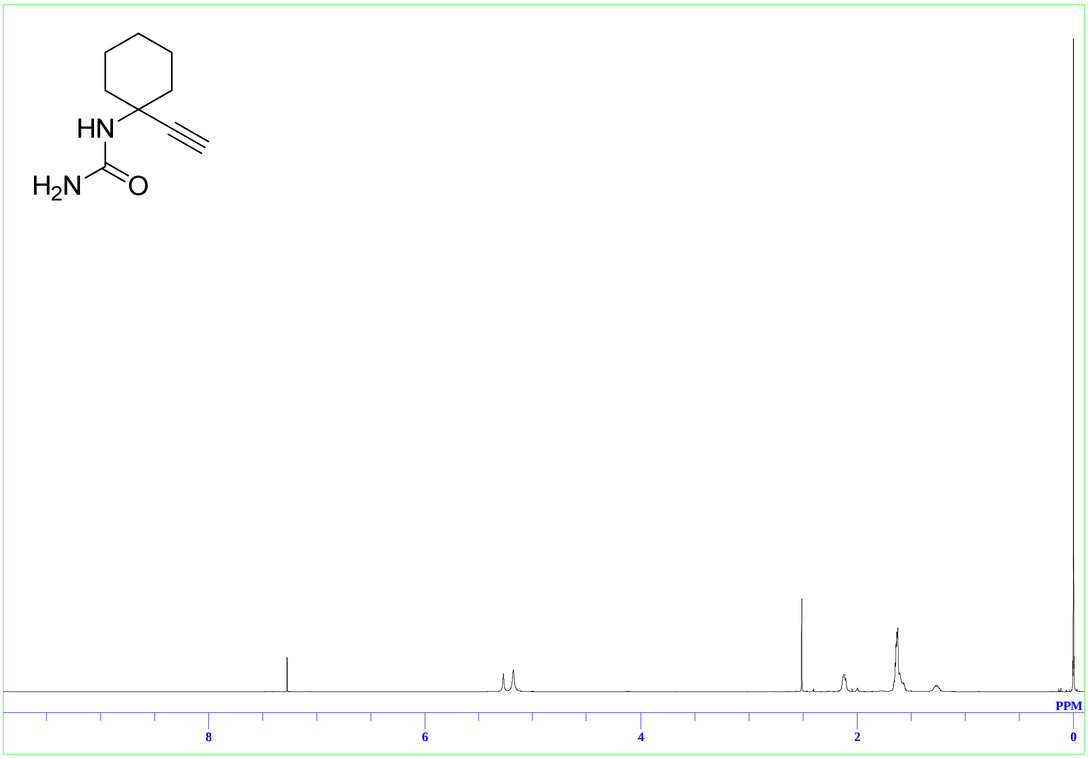

1i carbon

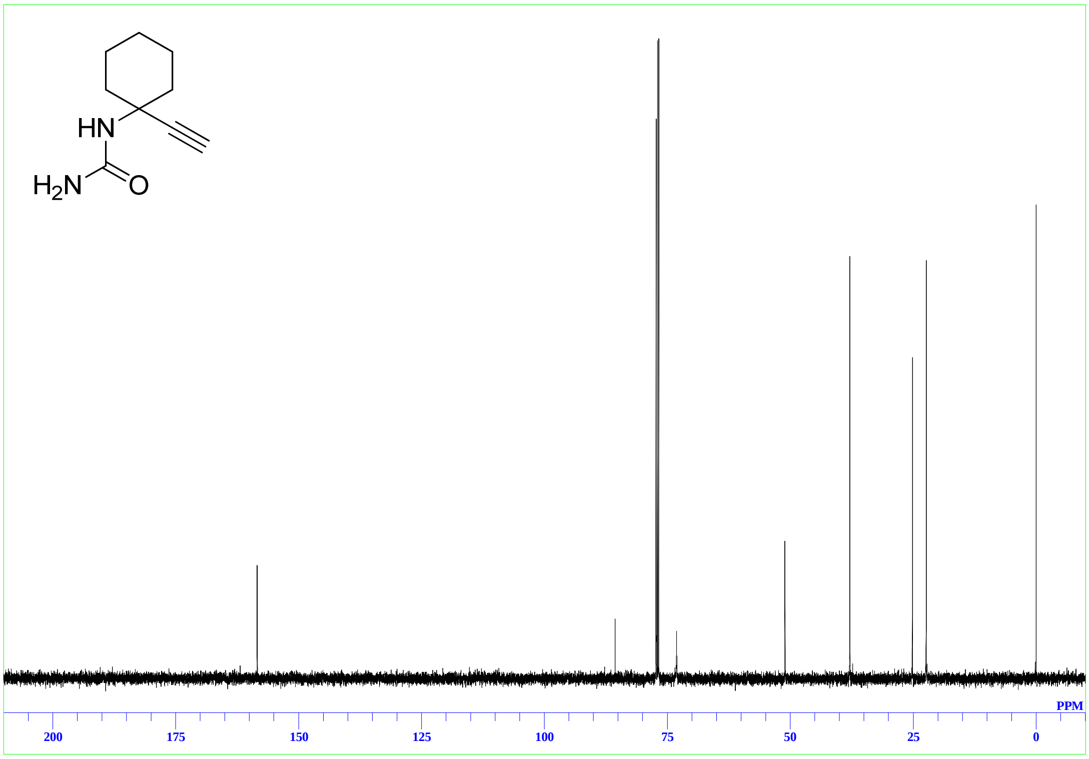

1j proton

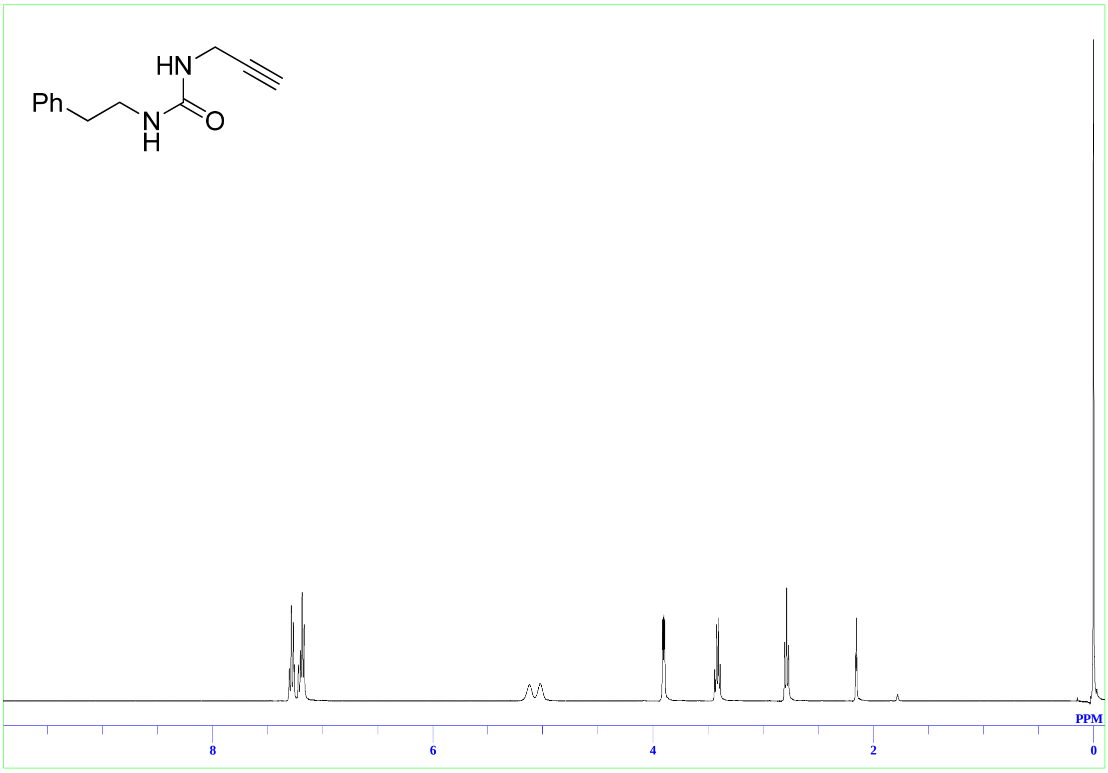

1j carbon

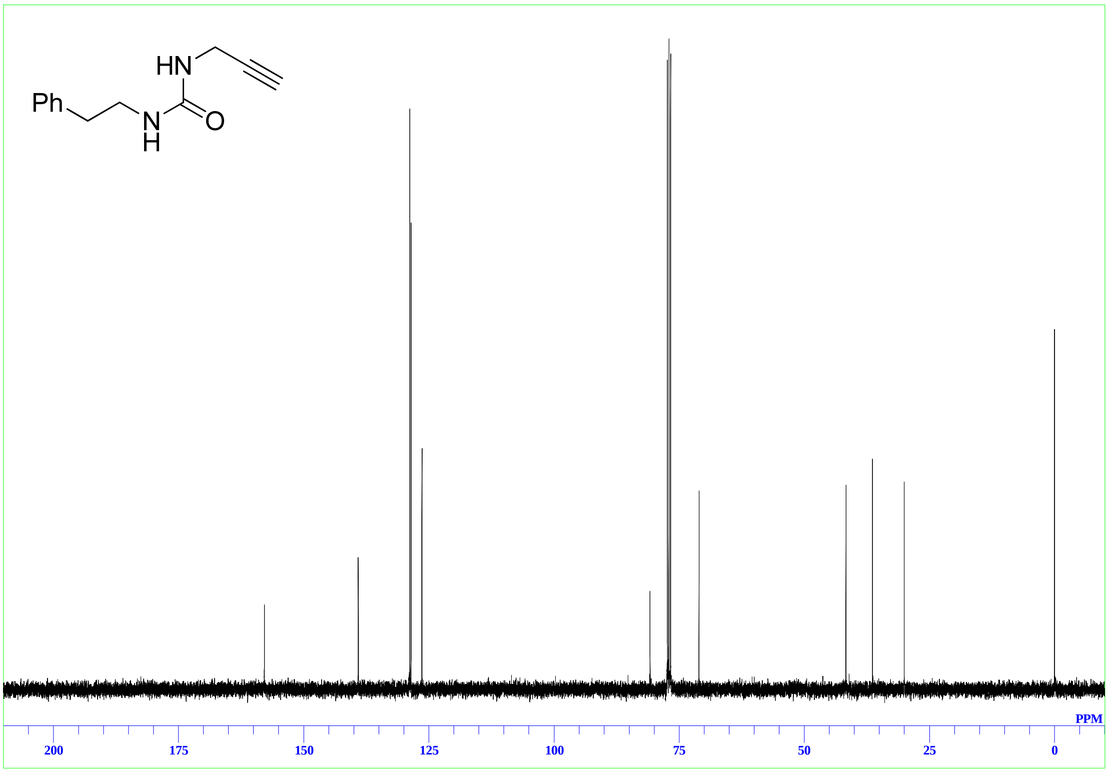

4a proton

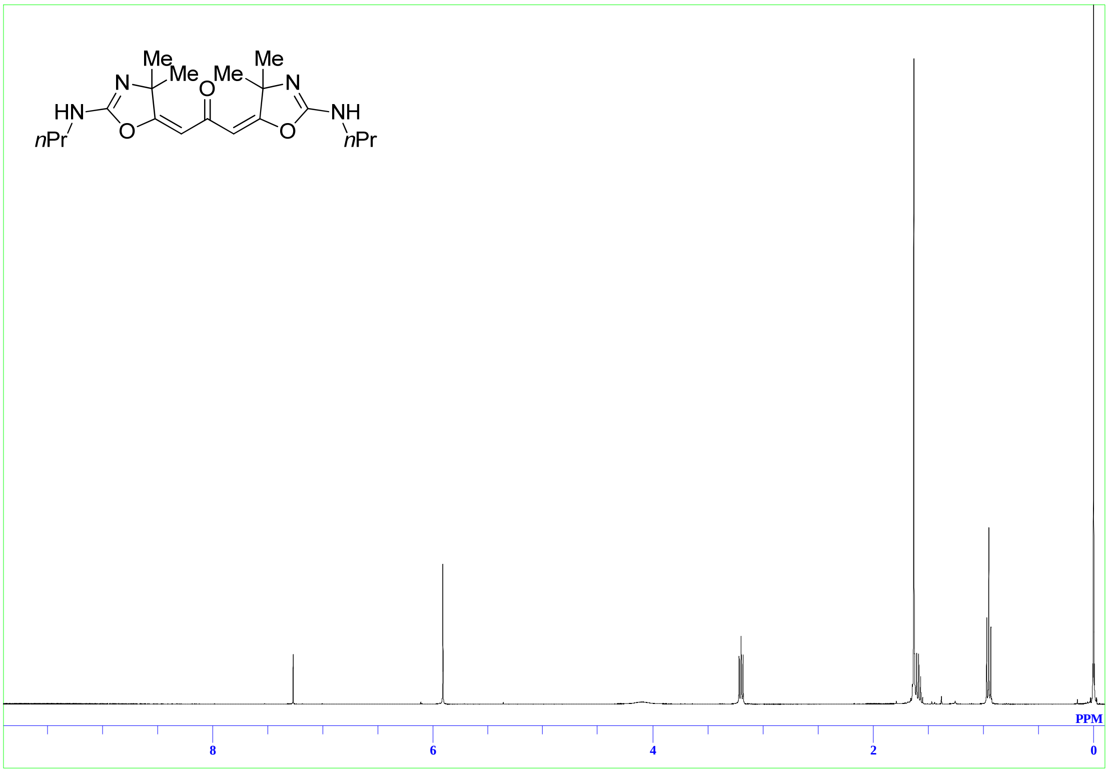

4a carbon

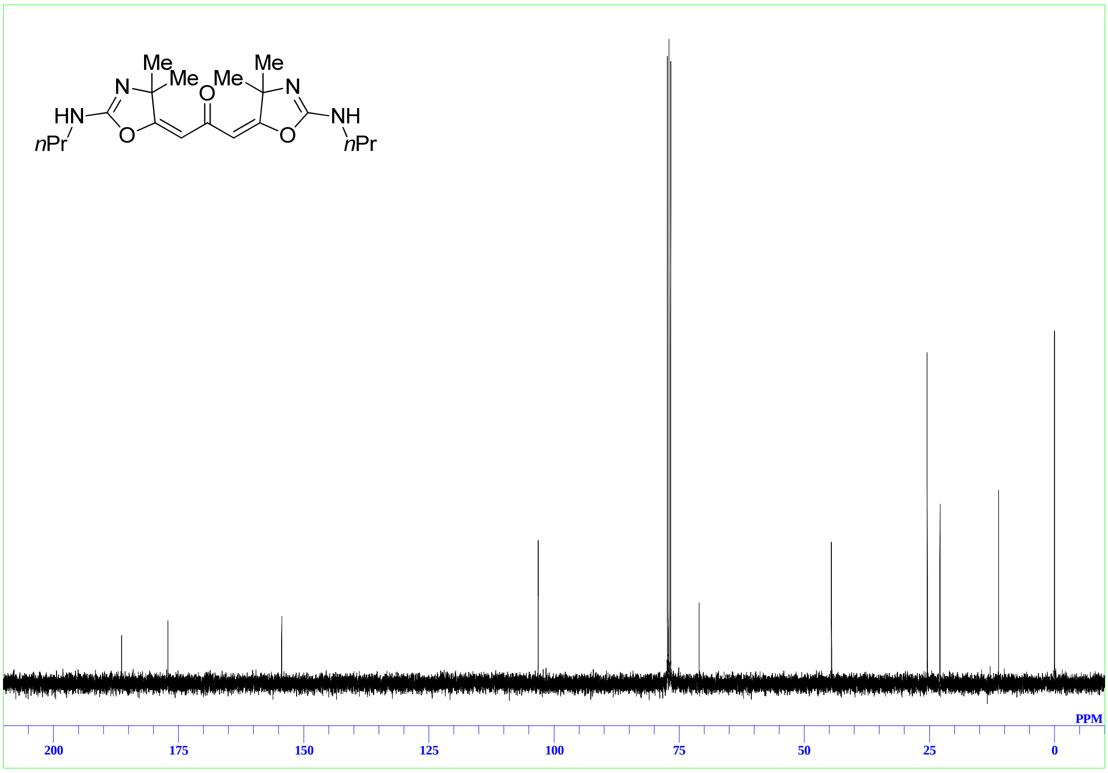

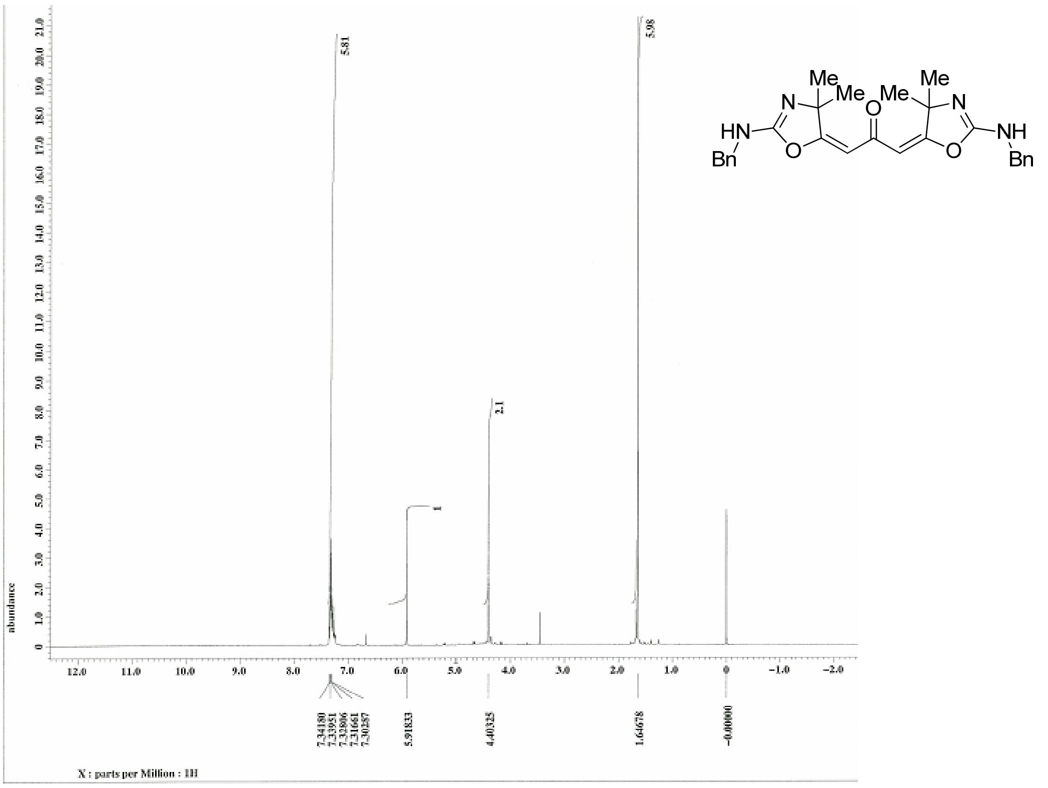
Me
Me
Me
Me
N
N
O
HN
NH
Bn
Bn
O
O
5.81
2.1
1
5.98
abundance
7.34180
7.33951
7.32806
7.31661
7.30287
5.91833
4.40325
1.64678
-0.00000
X : parts per Million : 1H

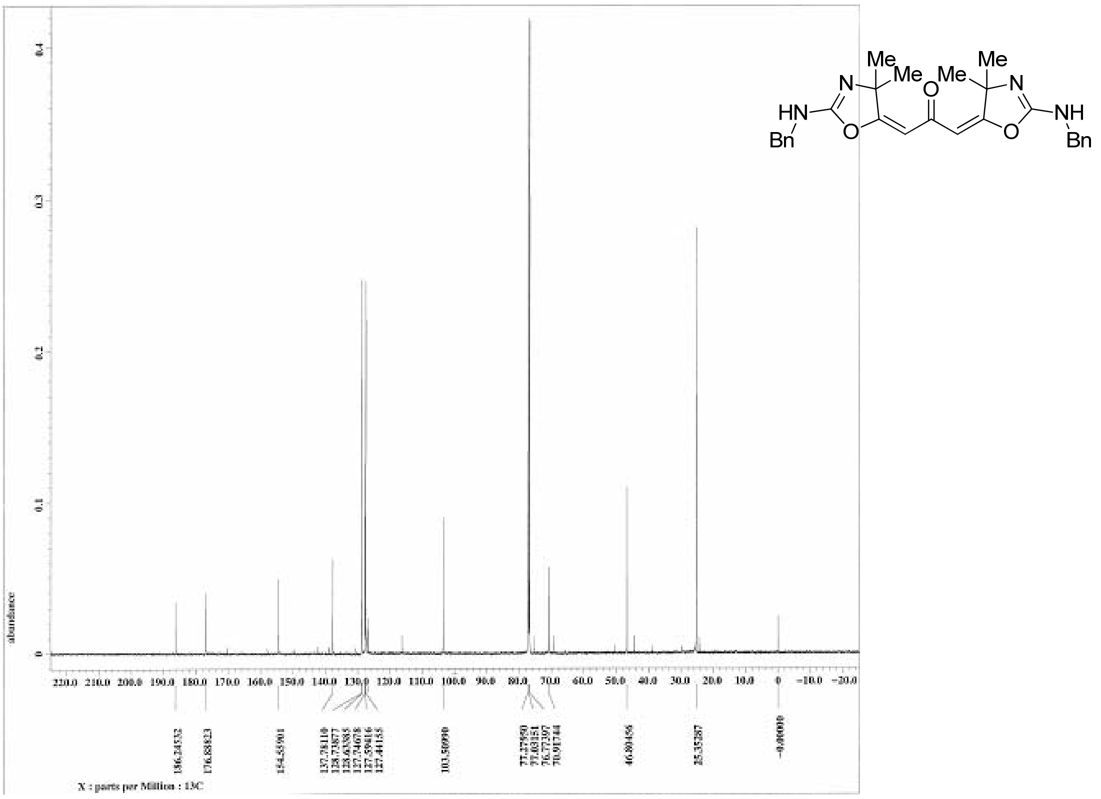
Me
Me
Me
Me
N
N
O
HN
NH
Bn
Bn
O
O
abundance
186.24532
176.83823
154.55901
137.78110
128.73877
128.63385
127.74678
127.59416
127.44155
103.50990
77.47950
77.03151
76.77307
70.91744
46.80456
25.35267
-0.00000
X : parts per Million : 13C

2c proton

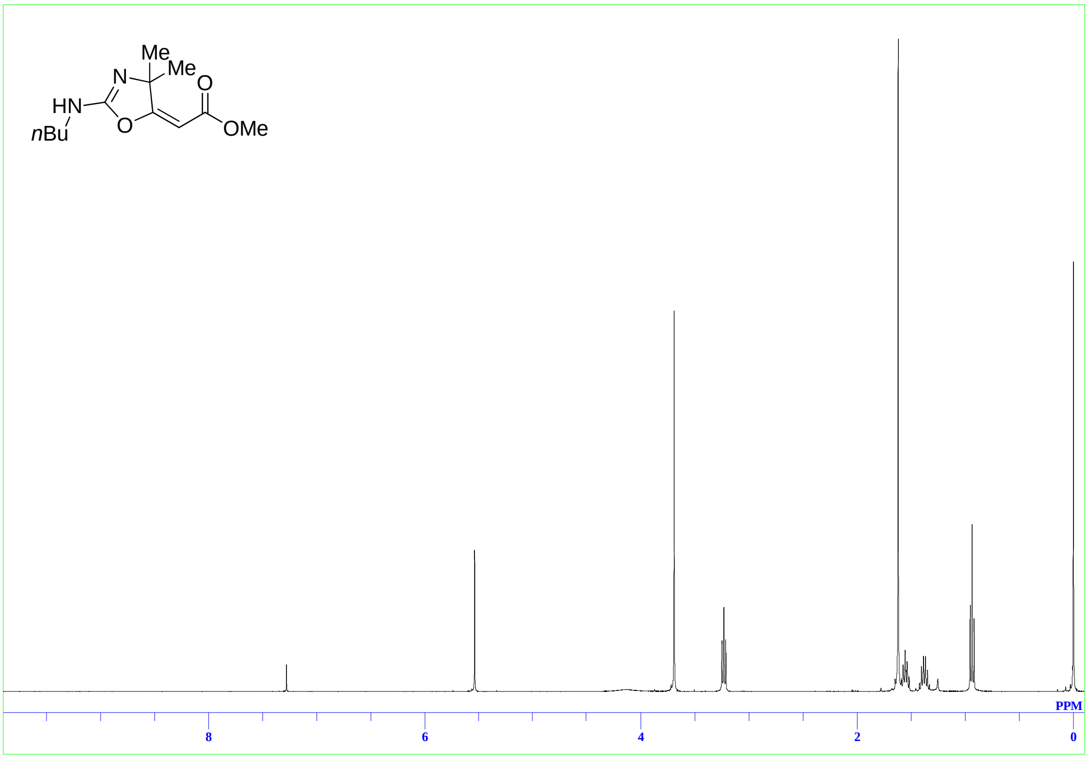

2c carbon

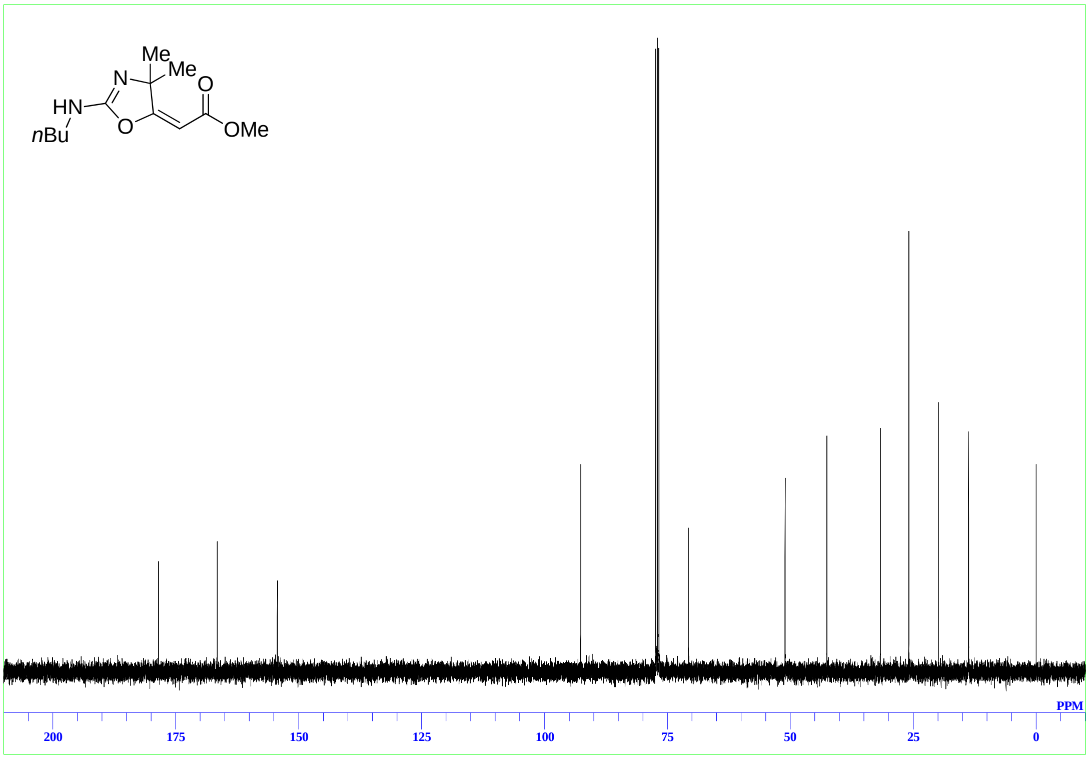

4c proton

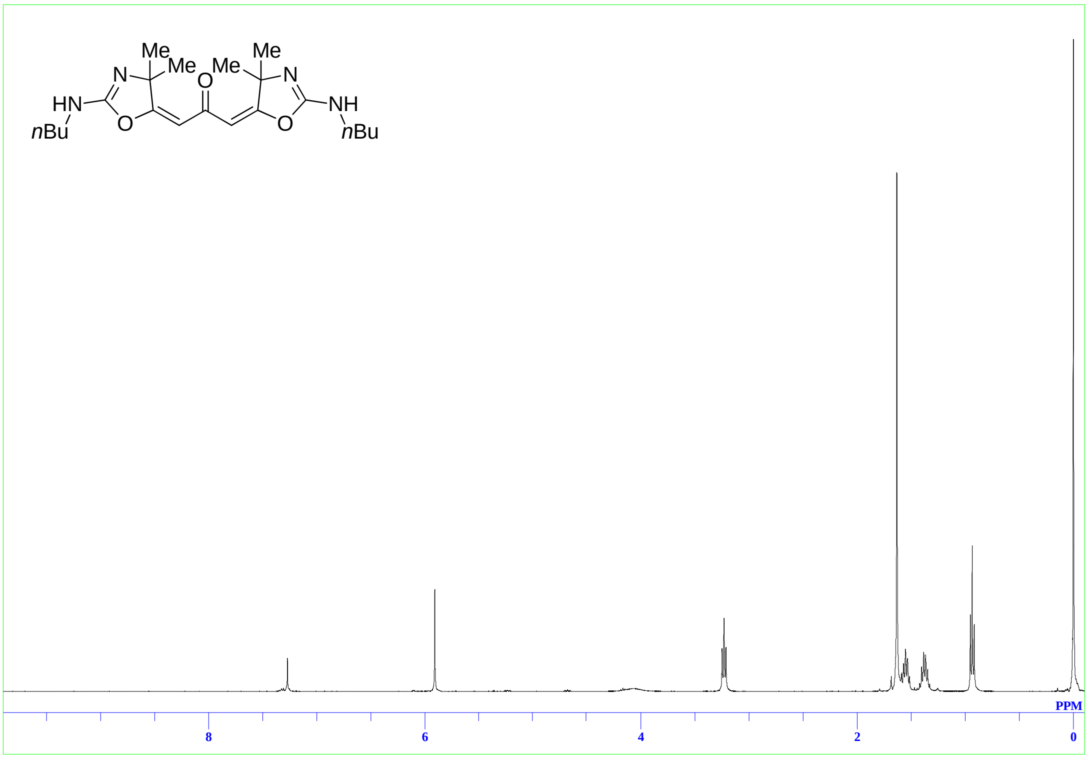

4c carbon

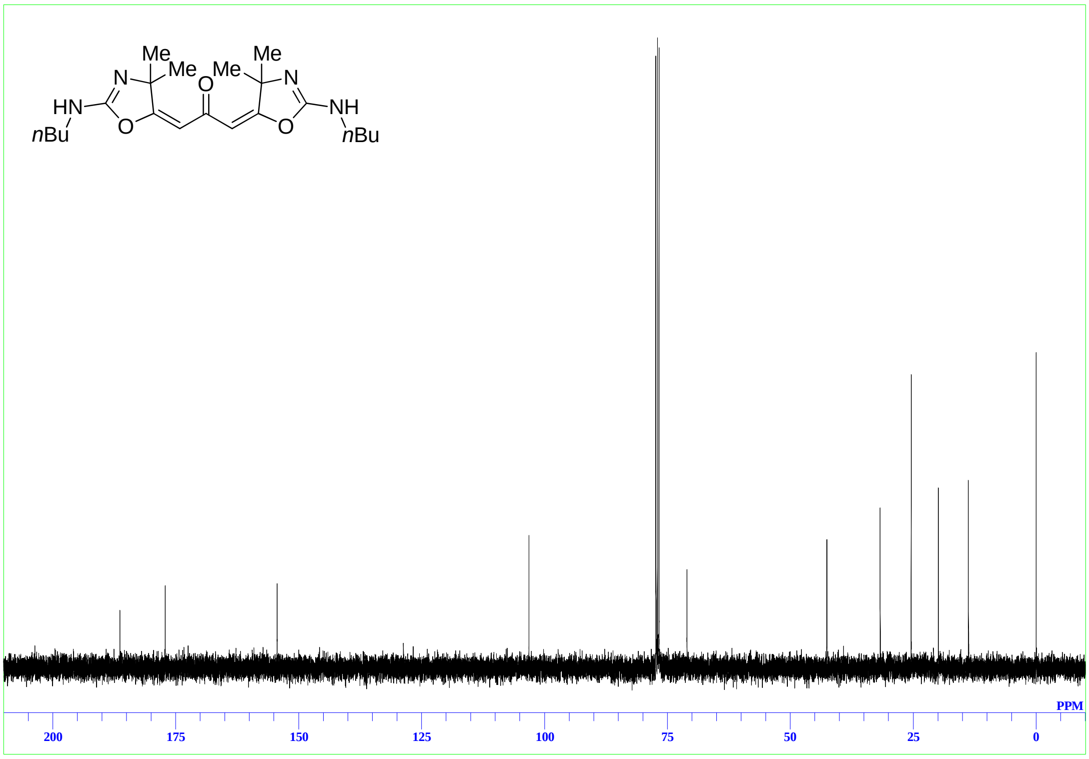

2d proton

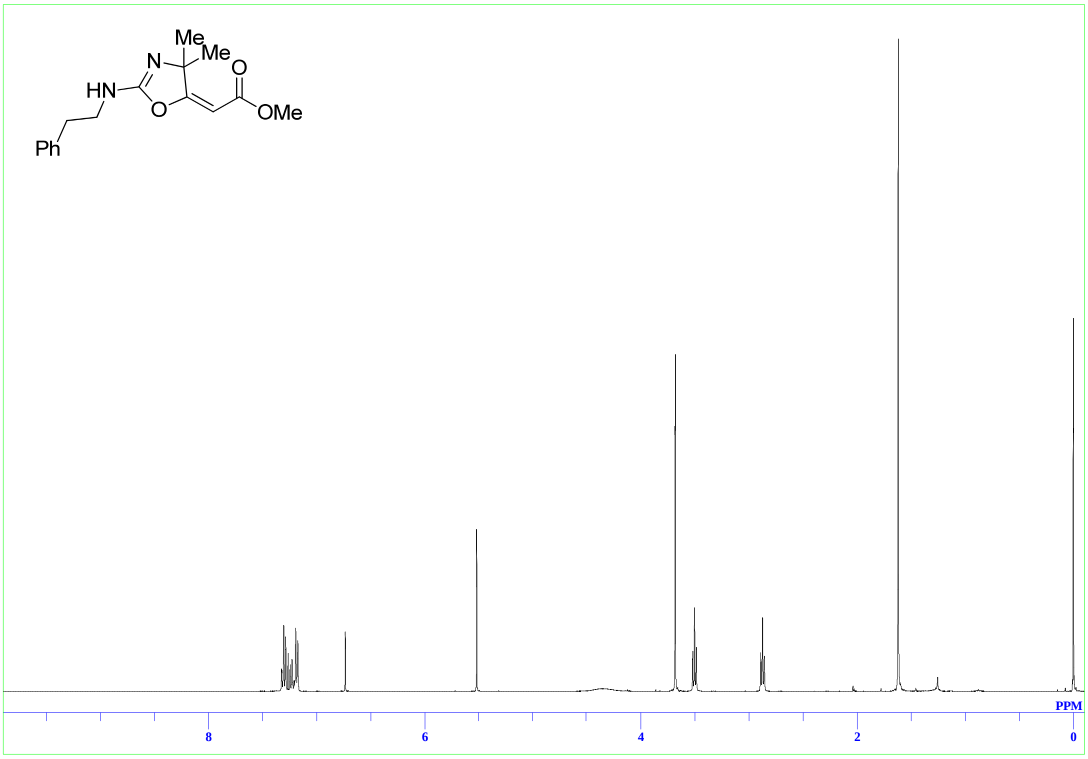

2d carbon

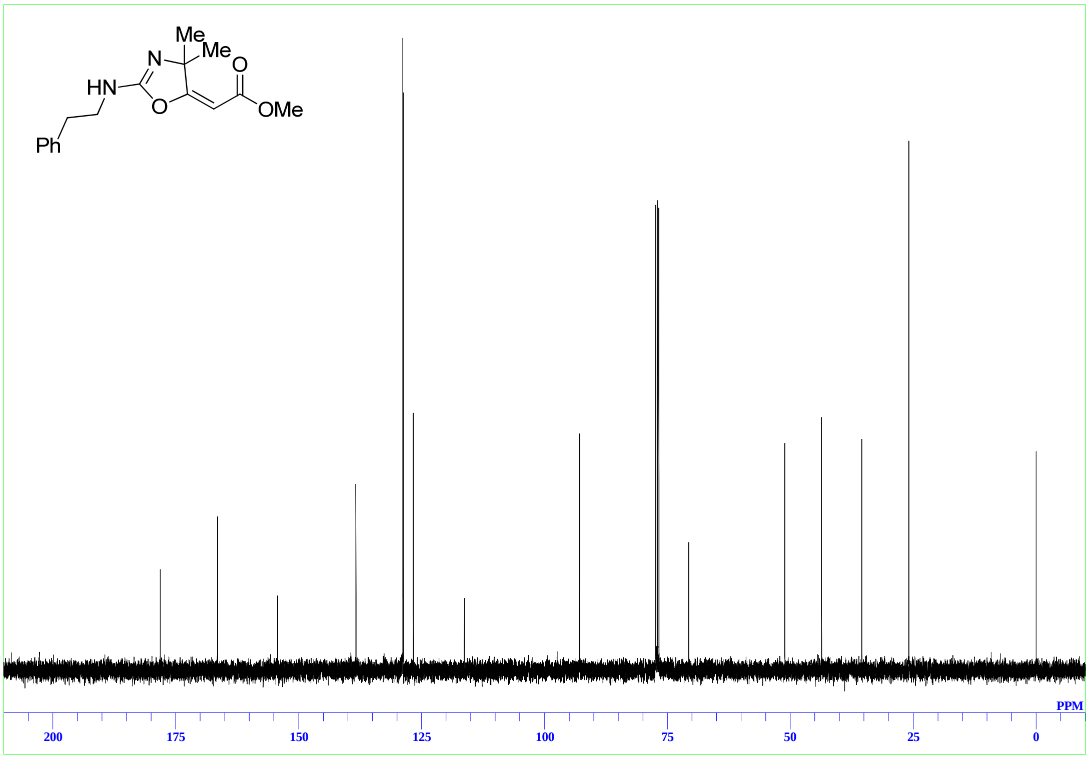

4d proton

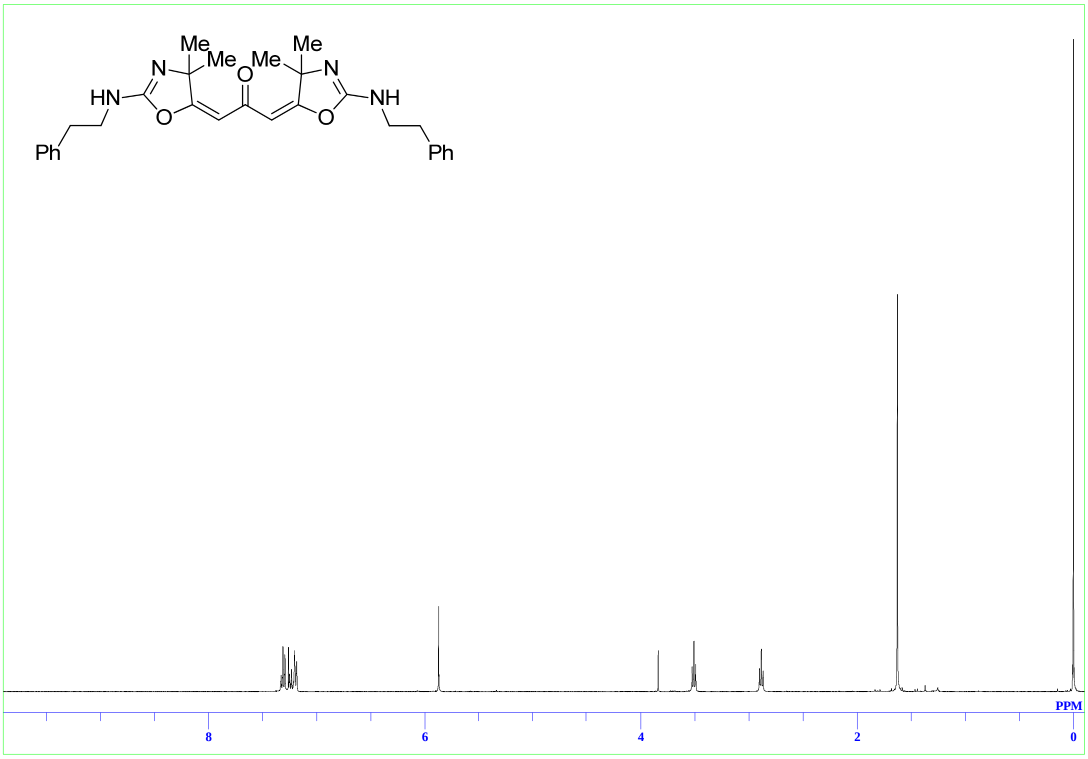

4d carbon

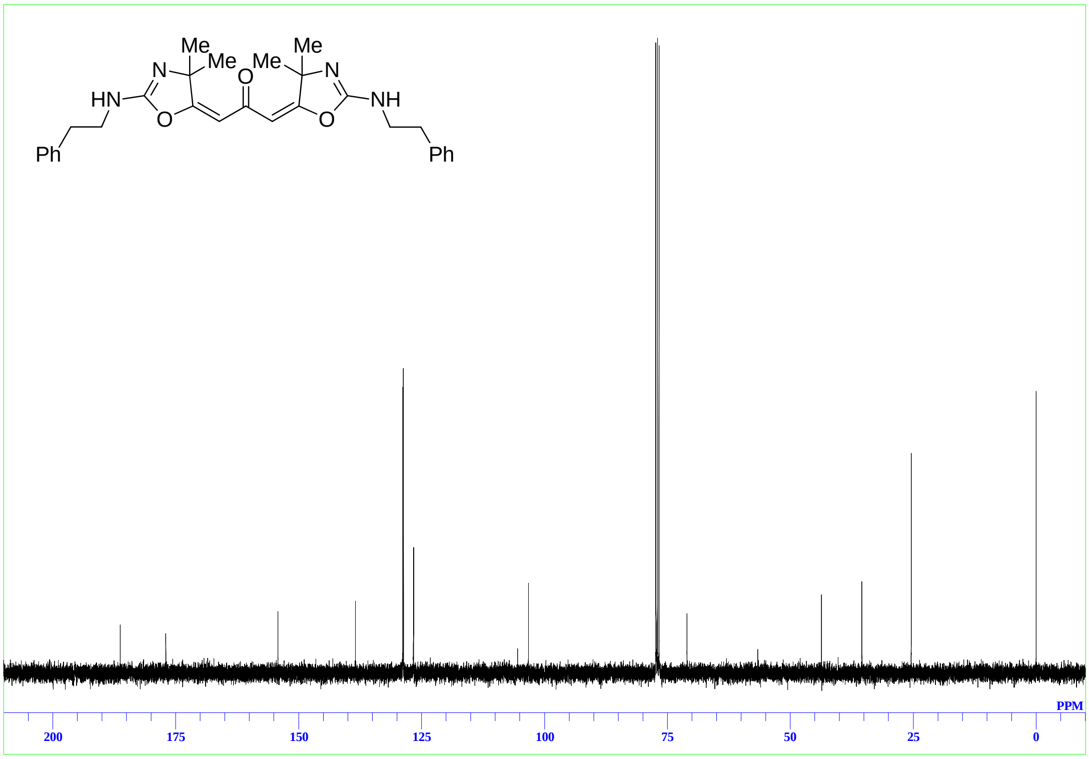

2e protpn

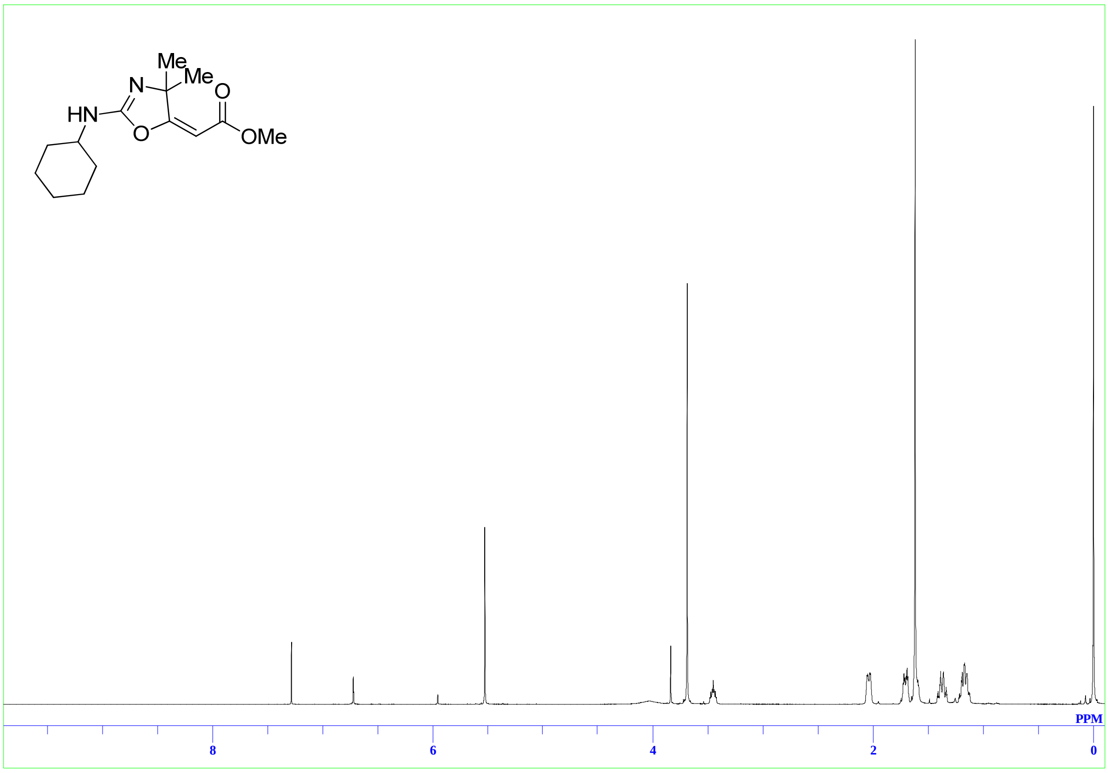

2e carbon

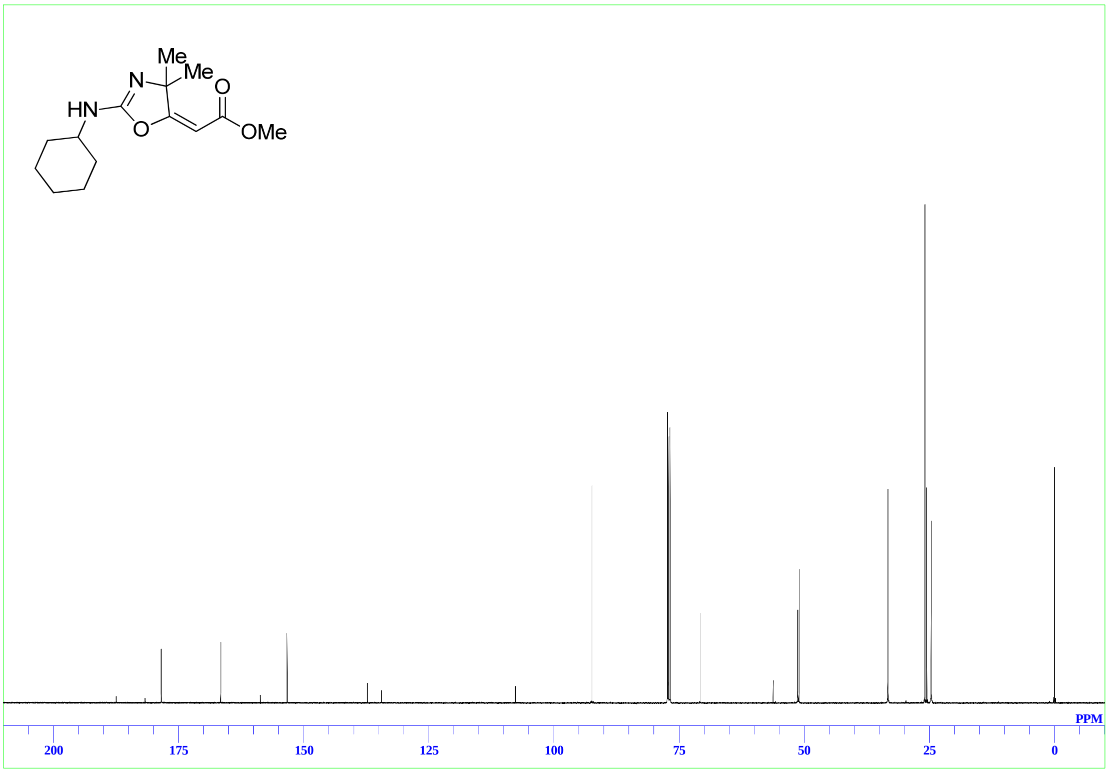

4e proton

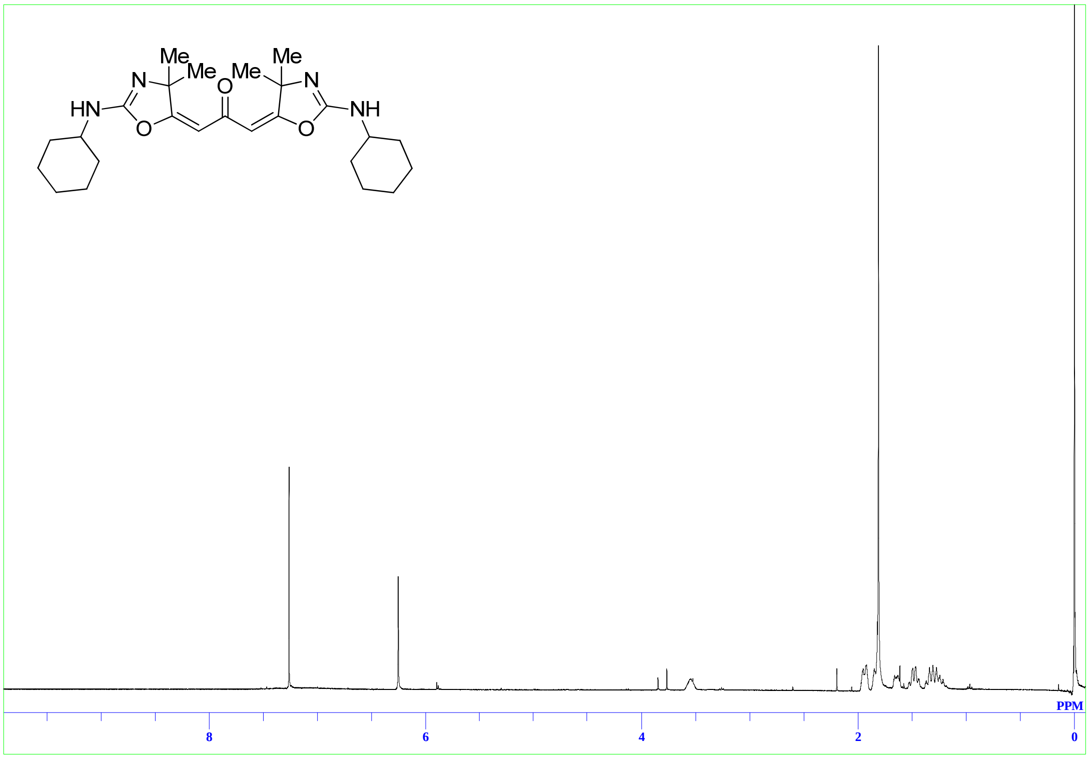

4e carbon

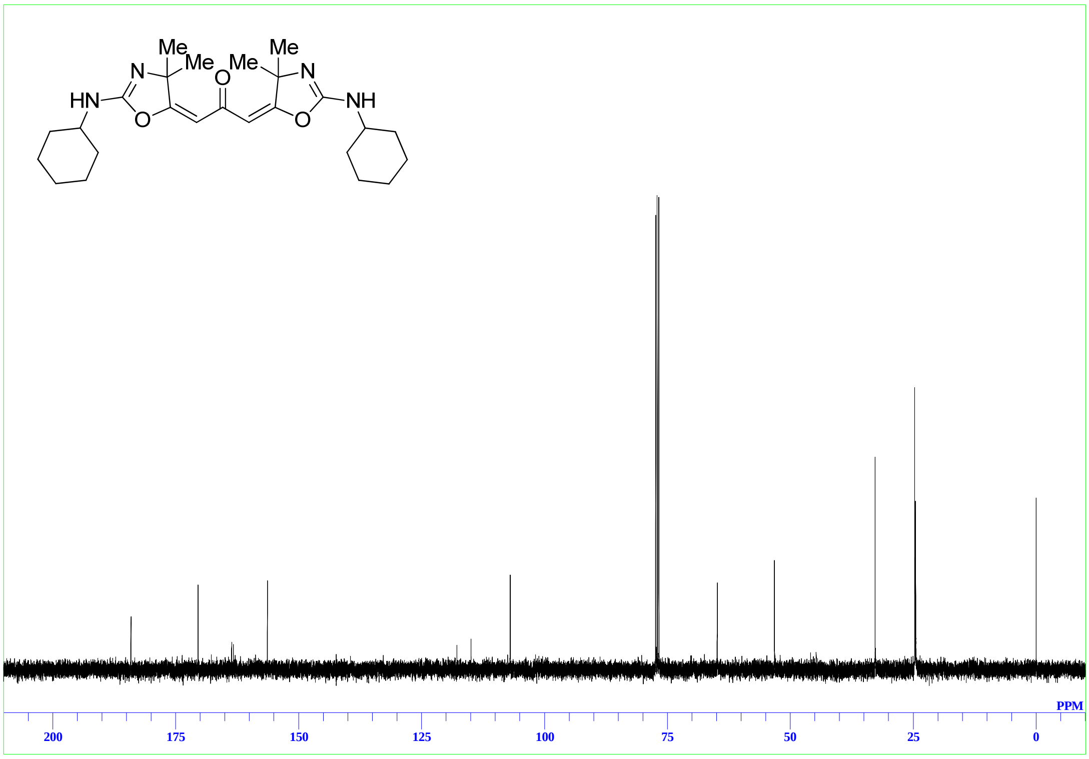

2f proton

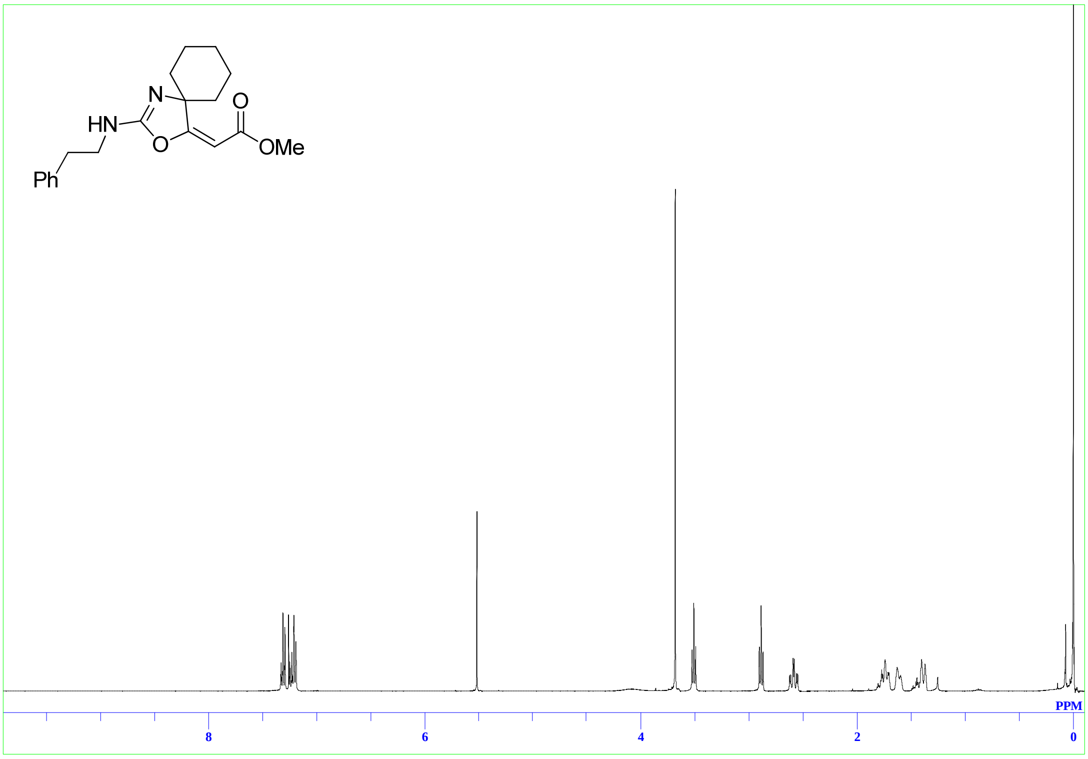

2f carbon

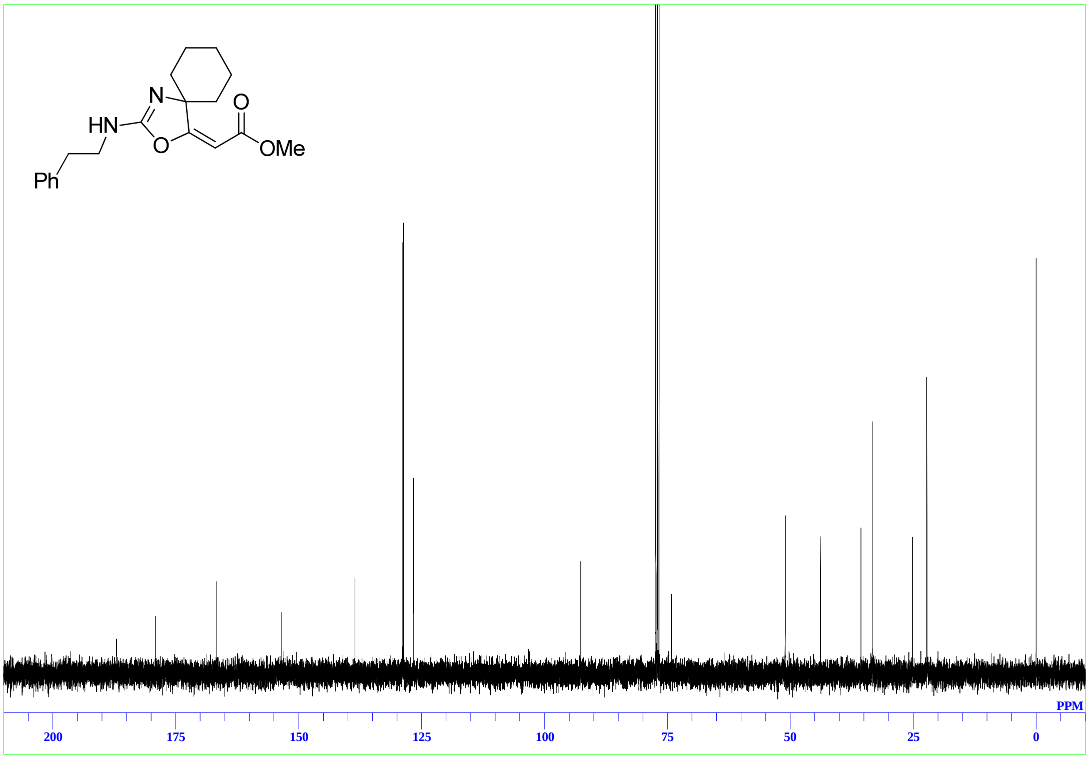

4f proton

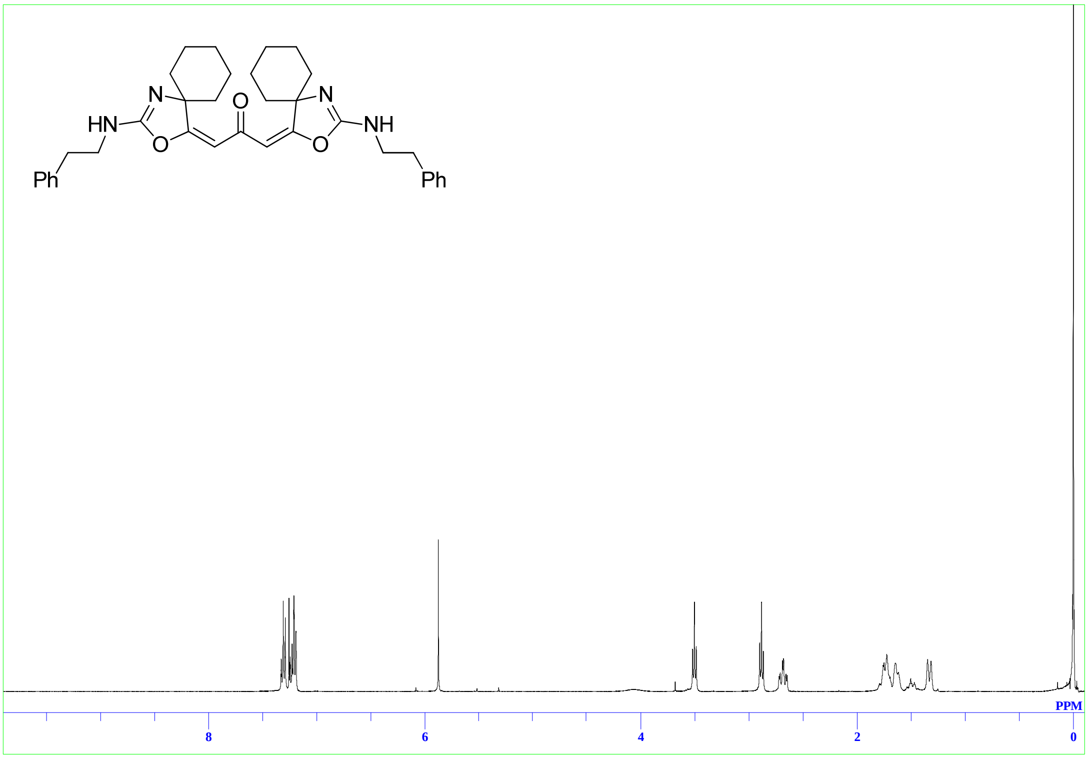

4f carbon

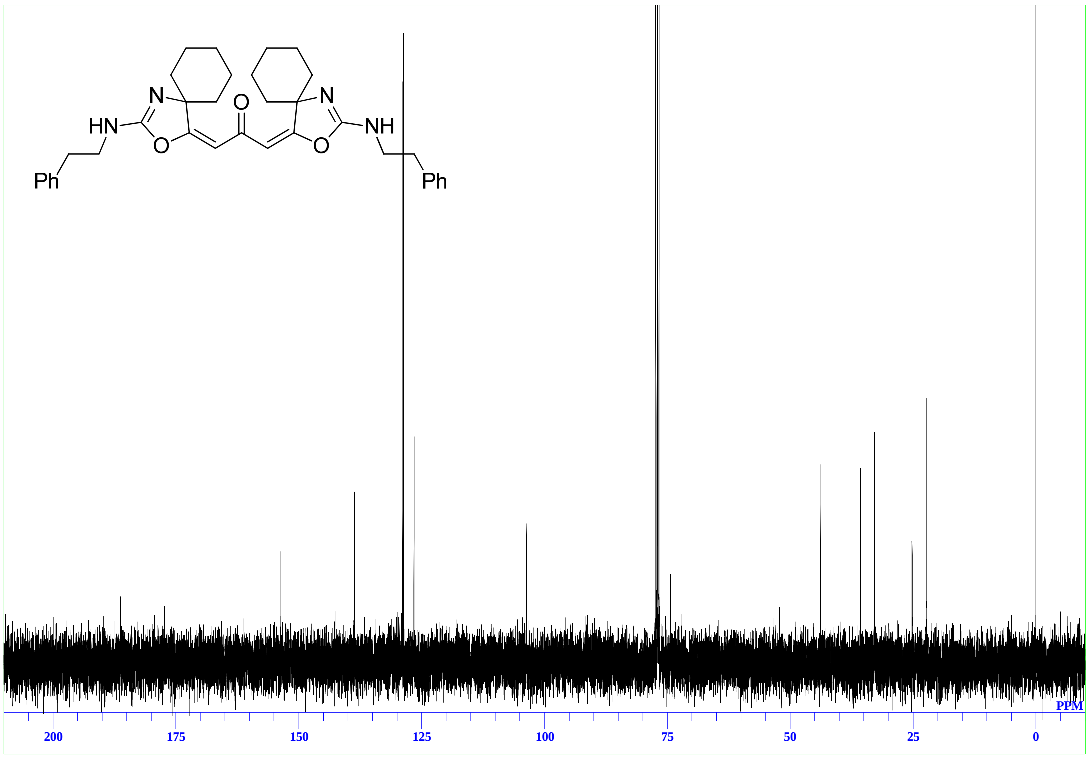

2g proton

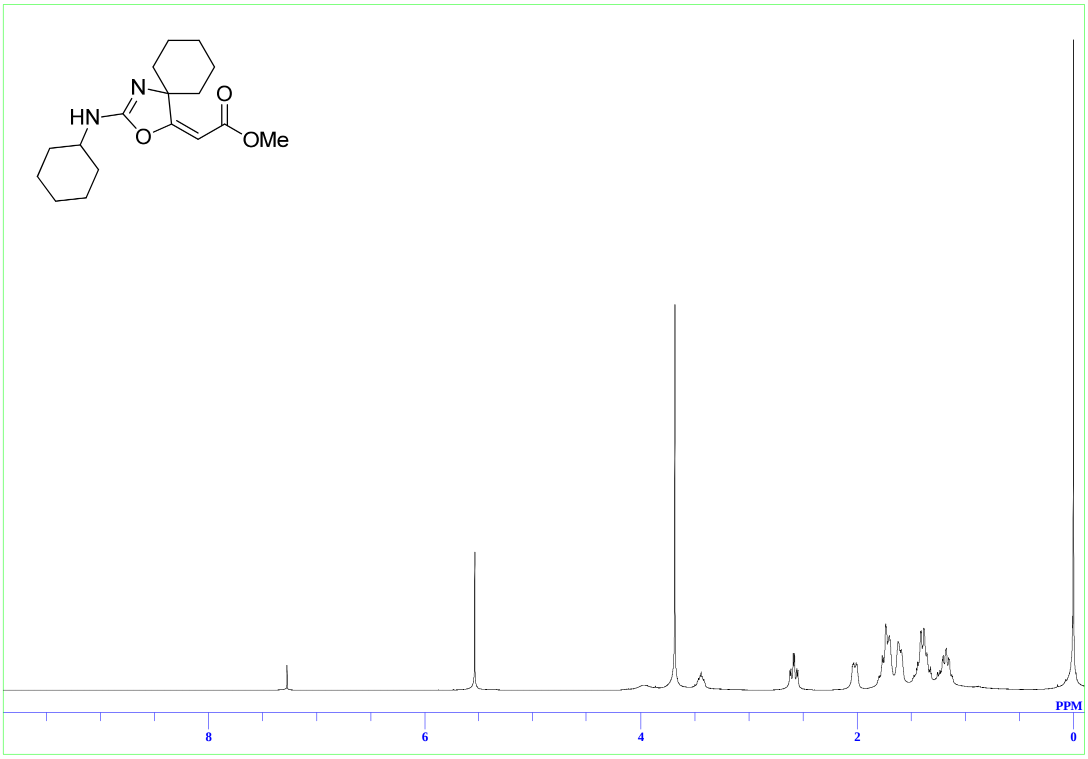

2g carbon

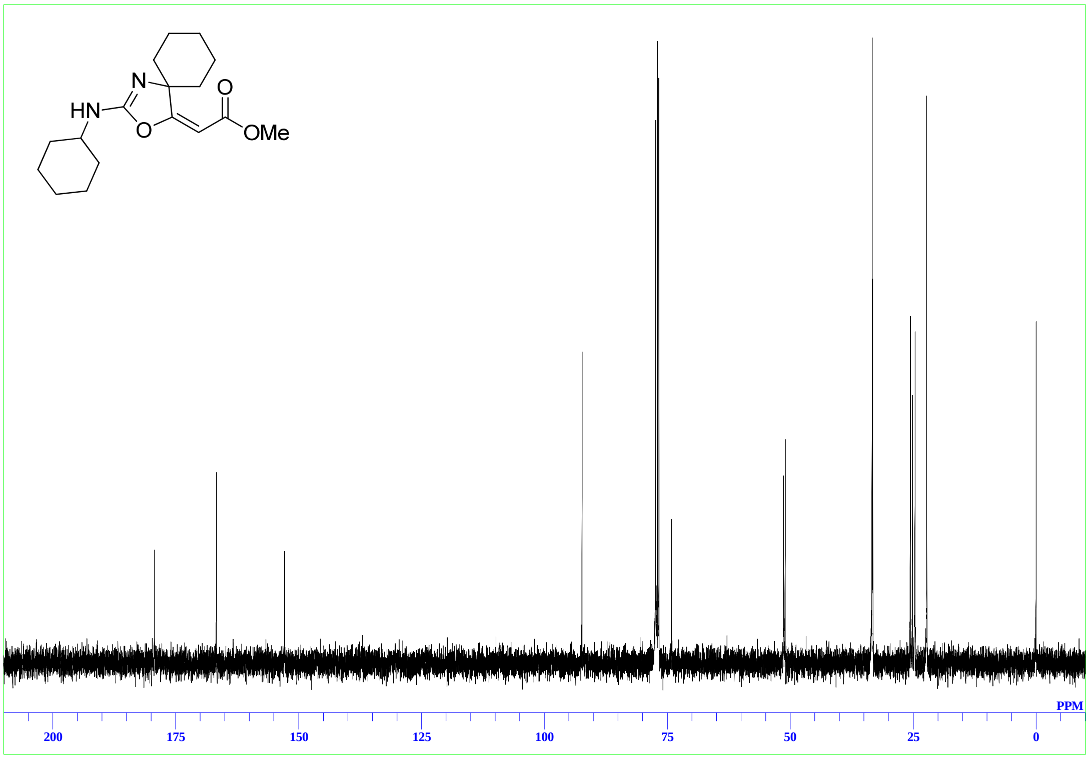

4g proton

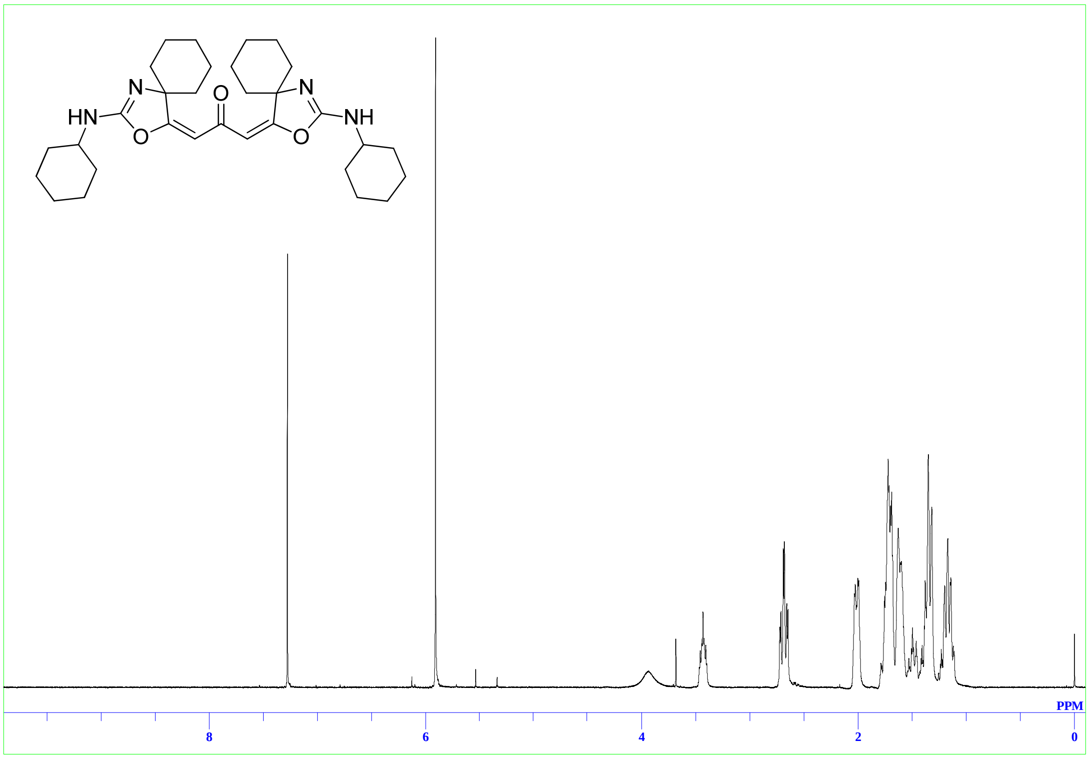

4g carbon

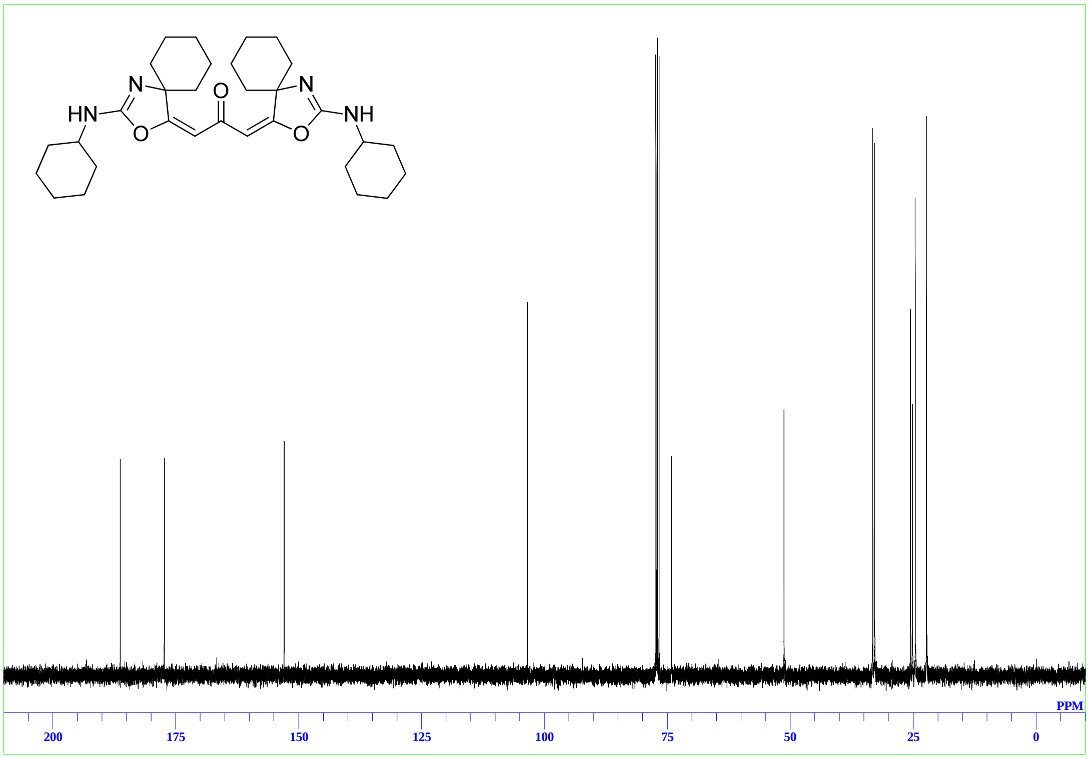

2g proton

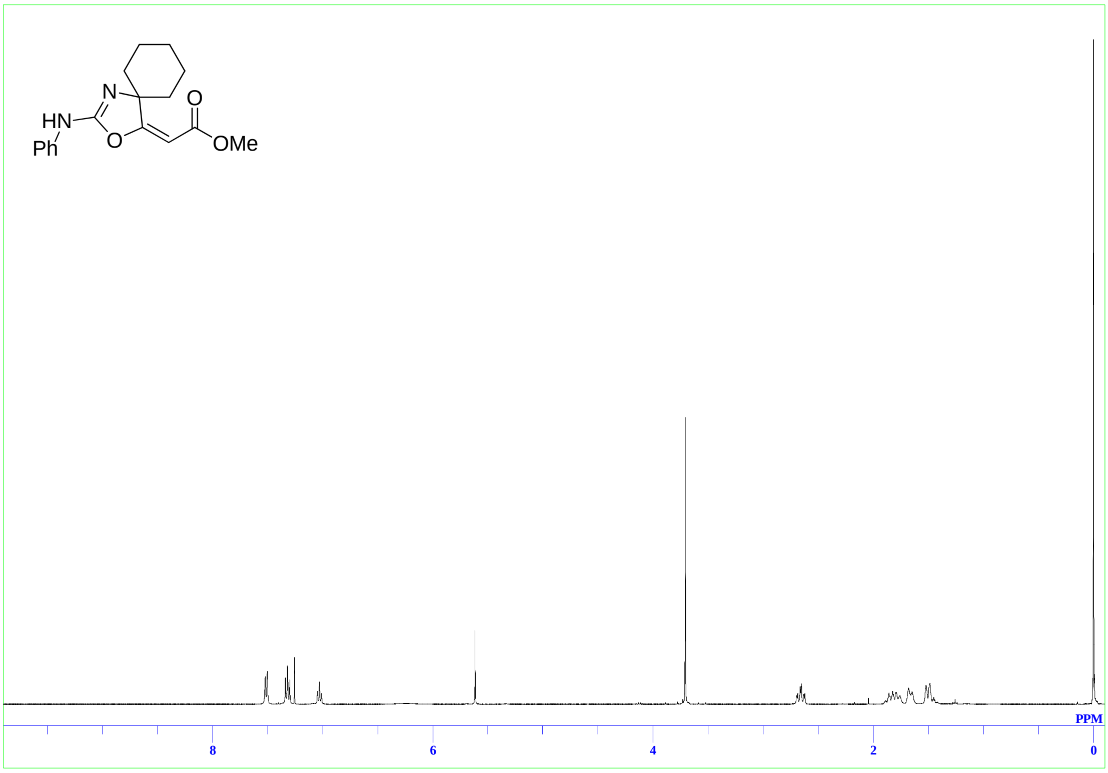

2h carbon

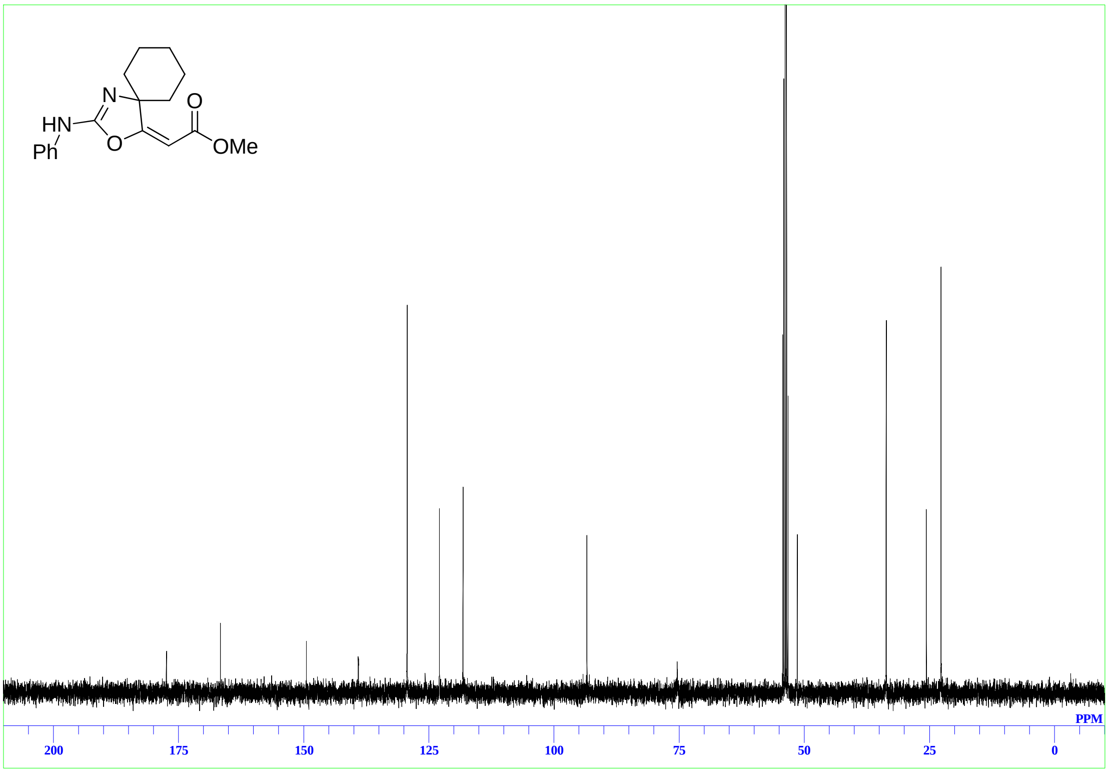

4h proton

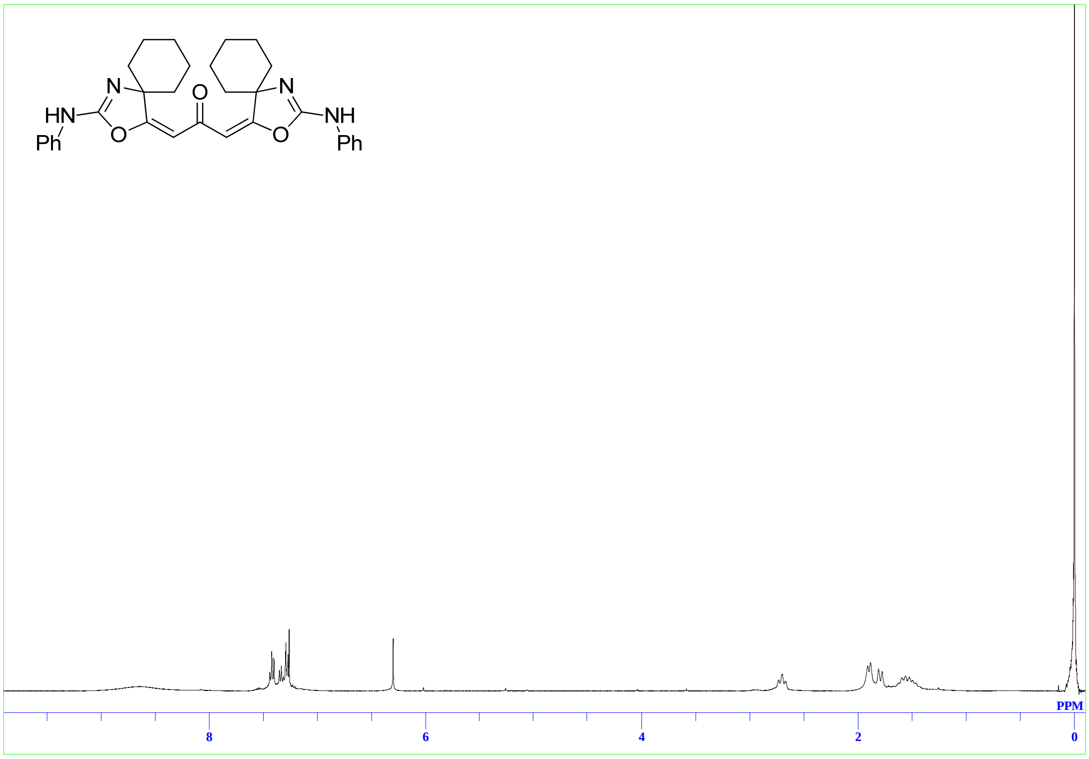

4h carbon

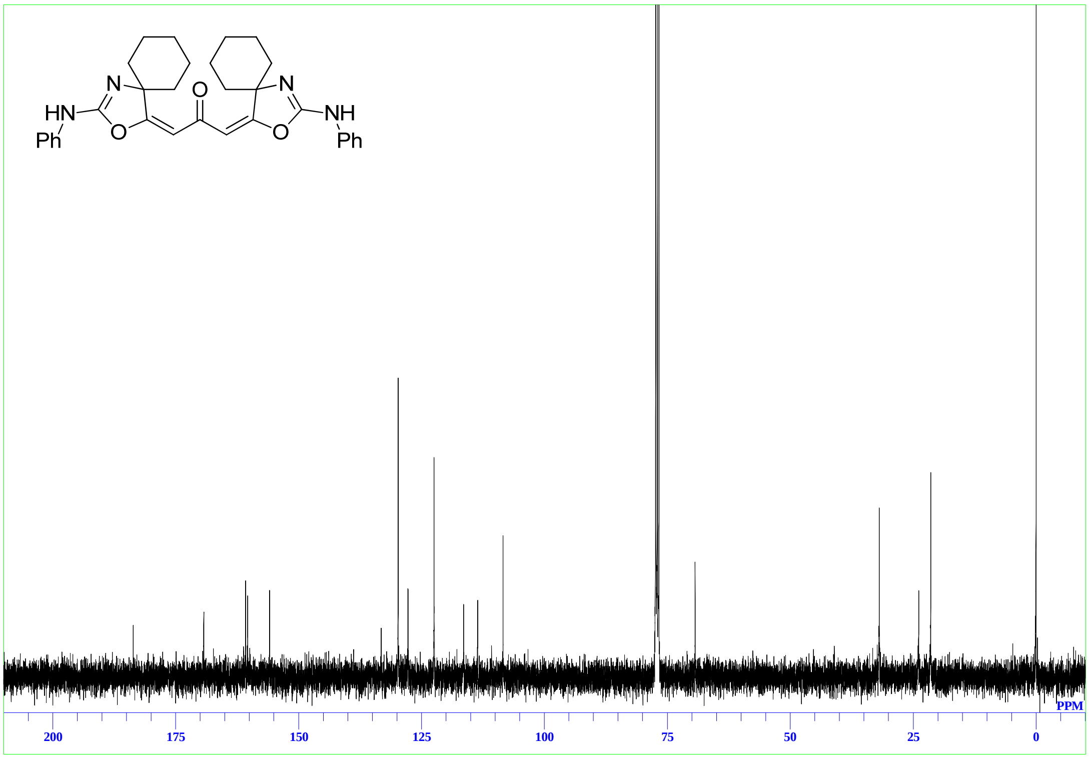

Supplement: Supplementary file 1 [file molecules-17-09220-s001.pdf]
